# Changes in salivary proteome before and after cigarette smoking in smokers compared to sham smoking in nonsmokers: A pilot study

**DOI:** 10.18332/tid/138336

**Published:** 2021-06-29

**Authors:** Indu Sinha, Jennifer Modesto, Nicolle M. Krebs, Anne E. Stanley, Vonn A. Walter, John P. Richie, Joshua E. Muscat, Raghu Sinha

**Affiliations:** 1 Department of Biochemistry and Molecular Biology, Penn State Cancer Institute, Hershey, United States; 2 Department of Public Health Sciences, Penn State Cancer Institute, Hershey, United States; 3 Mass Spectrometry and Proteomics Core, Penn State University College of Medicine, Hershey, United States

**Keywords:** proteomics, saliva, salivary alpha amylase, smokers, non-smokers

## Abstract

**INTRODUCTION:**

Smoking is the leading cause of preventable disease. Although smoking results in an acute effect of relaxation and positive mood through dopamine release, smoking is thought to increase stress symptoms such as heart rate and blood pressure from nicotine-induced effects on the HPA axis and increased cortisol. Despite the importance in understanding the mechanisms in smoking maintenance, little is known about the overall protein and physiological response to smoking. There may be multiple functions involved that if identified might help in improving methods for behavioral and pharmacological interventions. Therefore, our goal for this pilot study was to identify proteins in the saliva that change in response to an acute smoking event versus acute sham smoking event in smokers and non-smokers, respectively.

**METHODS:**

We employed the iTRAQ technique followed by Mass Spectrometry to identify differentially expressed proteins in saliva of smokers and non-smokers after smoking cigarettes and sham smoking, respectively. We also validated some of the salivary proteins by ELISA or western blotting. In addition, salivary cortisol and salivary amylase (sAA) activity were measured.

**RESULTS:**

In all, 484 salivary proteins were identified. Several proteins were elevated as well as decreased in smokers compared to non-smokers. Among these were proteins associated with stress response including fibrinogen alpha, cystatin A and sAA. Our investigation also highlights methodological considerations in study design, sampling and iTRAQ analysis.

**CONCLUSIONS:**

We suggest further investigation of other differentially expressed proteins in this study including ACBP, A2ML1, APOA4, BPIB1, BPIA2, CAH1, CAH6, CYTA, DSG1, EST1, GRP78, GSTO1, sAA, SAP, STAT, TCO1, and TGM3 that might assist in improving methods for behavioral and pharmacological interventions for smokers.

## INTRODUCTION

Smoking is the leading cause of premature mortality^1^. A major reason smokers have a difficult time quitting is the need to alleviate psychological stress and negative mood. Clinically, this is known as self-medication, where smoking is used to lower negative mood and stress, and increase positive mood. The behavioral reactions are rapid, as nicotine reaches the brain in a number of seconds. A single cigarette, which typically is only puffed about 10 times, is sufficient to temporarily satisfy smoking cravings and increase positive mood. Preclinical research also shows that stress increases drug self-administration and drug seeking behaviors. Some antidepressants facilitate smoking cessation but the mechanism is not known^2^. Little is known about the biology, psychology and the interplay between psychological and biological stress in response to smoking. There is a stress indication model of smoking, which postulates that soon after finishing a cigarette, smokers begin to experience adverse psychological symptoms such as changes in mood and stress associated with acute nicotine withdrawal^3^. The stress hormone cortisol is the standard biomarker of stress response, representing the activation of the hypothalamic–pituitary–adrenal (HPA) axis pathway. Both salivary amylase protein levels and sAA activity have also been used in stress research. Smoking related literature indicates impact on sAA activity as one of the stress related markers^4,5^. Given the limited effectiveness of standard of care treatment for nicotine dependence (e.g. counseling and nicotine pharmacotherapy), a greater understanding of the role of stress could potentially lead to new and improved methods for behavioral and pharmacological interventions.

The typical pack-a-day smoker waits approximately 60 minutes or less between each cigarette throughout awake-hours. The maintenance of smoking throughout the day is thought to reflect smokers’ attempts to regulate mood and feelings of relaxation.

The biological pathways in the maintenance of smoking from one cigarette to the next are not well understood. The one established mechanism is that smoking increases adrenocorticotropic hormone (ACTH), which stimulates cortisol secretion^6,7^. This response has been attributed to the nicotine in tobacco smoke^8^. In the drug dependence field, proteomics has been proposed to identify protein patterns that underlie psychological and neurological mechanisms^9^. A study demonstrated rapid effects of stress-induced HPA activation in rats, following only a 15-minute physical stressor^10^. Hwang and Li^11^ identified several differentially expressed proteins in various brain regions of rats administered with nicotine. Cecconi et al.^12^ observed significant changes in C-reactive protein along with other serum proteins in rats at different time points during nicotine administration and nicotine extinction cycles within a 2-week period. Rat models have also been recently employed to study secondhand smoke exposure^13^.

There are mixed findings on smoking status and cortisol. The Steptoe et al.^14^ study found smoking is associated with an acute increase in cortisol, and 6-week abstinence reduced cortisol. Although it has been reported for decades that smoking helps reduce negative affect, the effect is temporary whereas smoking increases the heart rate, which increases anxiety, a physiological component of the stress response^15^. One study showed that long-term quitters had reduced perceived stress levels compared to non-quitters^16^. The application of proteomics to understanding nicotine dependence is virtually unexplored. Nicotine dependence is thought to be due to the moods (e.g. relief of stress, anxiety, etc.) associated with nicotine intake^6^. While a number of studies have investigated the effects of nicotine on a specific gene or protein response, in either *in vivo* or *in vitro* systems^9^, systematic studies on proteomic profiles during chronic exposure to nicotine have not been conducted and few studies on nicotine consumption in free living smokers have been reported. Moreover, sAA exhibits a stable circadian pattern that mirrors that of salivary cortisol, and has the potential to be accepted as a non-invasive biomarker for the sympathetic nervous system (SNS)^17,18^. However, there are few data on how it is influenced by smoking.

Several biological fluids have been used for measurement of proteins in smokers including plasma^19^, sputum^20,21^, bronchoalveolar lavage fluid^22,23^, and saliva^24,25^. We have also used exhaled breath condensate (EBC) for lung proteomics^26^ but EBC and BALF are methods suited for studying proteins in the respiratory tract. In addition, cortisol is a key marker for the purposes of this study, which can be obtained from saliva but not EBC. Saliva is also considered an easily obtainable clear fluid, which is indicative of individual’s profile at the time of collection. For research subjects, the non-invasive collection techniques of saliva dramatically reduce the anxiety and discomfort and simplify procurement of repeated samples for monitoring overtime.

The goal of the current pilot study was to determine the feasibility of using proteomics in human subjects’ research to identify new protein markers in response to acute smoking. There is a model for studying the acute effects of cigarette smoke on biological responses *in vitro*^27^ and in humans^28^. We took a similar approach for the current pilot study and analyzed saliva samples from current smokers and non-smokers before and after smoking two cigarettes/sham smoking two cigarettes, respectively, with a gap of 60 min between smoking sessions, using isobaric tags for relative and absolute quantitation (iTRAQ) methodology to identify new proteins that might assist in understanding changes that occur in the stress response to smoking.

## METHODS

### Participants

Smokers included three males and two females (aged 19–55 years) that had previously participated in the Pennsylvania Adult Smoking Study^29^. Inclusion criteria for this pilot study were: smokers, aged ≥18 years and smoked at least 1 cigarette (Cig) per day for the past year; non-smokers, aged ≥18 years and not smoked in the past 2 years. Exclusion criterion was: being pregnant. All smokers were Caucasian with less than college education and participants smoked between 15–30 cigarettes per day. Four control samples were obtained from non-smoking employees aged 19–55 years including two men and two women. All were college educated. Three were Caucasian and one was Asian. All the participants signed a consent form prior to their participation in the pilot study. The study was conducted in accordance with the Declaration of Helsinki, and the protocol was approved by the Institutional Review Board (IRB# PRAMS042265EP) of Penn State University College of Medicine.

### Procedures

All participants entered the study by completing a telephone interview that determined eligibility and were provided a description of the study. Participants who were eligible and interested were scheduled for one study visit at home for smokers. For non-smoker participants the visit was hosted within the laboratory setting of Penn State College of Medicine. At the visit, participants gave written consent and completed interviewer-administered questionnaires. The smokers were asked to smoke their usual brand of cigarette and after a mouthwash they were asked to chew on flavor-free gum; 6–8 mL of saliva was collected, by spitting, into a glass Pasteur pipette in a DNAse-RNAse-free polypropylene tube containing 1 mM sodium orthovanadate and protease inhibitors (Sigma-Aldrich, St. Louis, MO) and placed on ice. The smoking session was repeated after a gap of at least 1 h and tubes from both collection times were stored on ice for 1–2 h at which point, tubes were transported to the laboratory for further processing. Non-smokers were asked to use a sham unlit cigarette and perform the saliva collection as described above. Saliva samples were collected from 5 smokers and 4 non-smokers before and 30 minutes after each smoking session. Saliva tubes were centrifuged at 10000 rpm for 10 min at 4^o^C and separate aliquots of supernatants were stored frozen at -80^o^C until analysis. Most studies of changes in reported stress level have looked at the effects of withdrawal over a long time period. We chose to go with an acute time frame of 30 min after smoking. All studies started at about 12:00–12:30 p.m., to account for the diurnal variation in cortisol levels, which spike in the early morning hours.

### Sample processing for iTRAQ analysis

For the iTRAQ analysis (Supplementary file Figure S1), 5 saliva samples were pooled from smokers separately at baseline and after smoking the first cigarette (Cig 1). Similarly, saliva samples before and after smoking the second cigarette (Cig 2) were also pooled separately. In addition, 4 saliva samples were pooled from non-smokers before and after first sham unlit cigarette smoking separately and before and after second sham cigarette separately for a total of 8-pooled samples. All the samples were processed according to the Penn State University College of Medicine (PSUCOM), Mass Spectrometry and Proteomics Core Facility protocol adapted from the manufacturer’s instructions (AB SCIEX, Framingham, MA) (http://med.psu.edu/web/core/proteinsmassspectometry/protocols/itraq) as described earlier^30^. Briefly, the iTRAQ methodology involved digesting equal amounts of protein from the pooled samples (100 μg) with trypsin and subsequently labeling each pooled sample with a different tag (Supplementary file Figure S1). The 8 different isobaric tags add the same mass to primary amine groups in the tryptic peptides from each pooled sample, but with each tag composed of different proportions of 12C→13C and 14N→15N substitutions in one portion of the tag, yielding quantitative fragments ranging from 113 to 121 Daltons upon MS/MS fragmentation. The amount of each of these fragments arising from fragmentation of each peptide peak shows proportionally how much of each peptide peak came from each individually labeled sample. The labeling with tags was performed using the iTRAQ^®^ Reagent-8Plex Multiplex kit (AB SCIEX, Framingham, MA). These tagged samples were submitted to the Proteomic Core Facility at PSUCOM. The labeled peptides were then resolved by two-dimensional liquid chromatography prior to matrix-assisted laser desorption and ionization time-of-flight tandem mass spectrometry. The details on the separation and analysis of proteins by 2D-LC as well as the methodology involving mass spectroscopy (MALDI TOF/TOF) are described in the Supplementary file. Peptide identification, protein grouping, and subsequent protein quantitation were done using the Paragon and ProGroup Algorithms in the ProteinPilot™ 4.5 Software package (AB SCIEX, Framingham, MA), searching the NCBI human database plus a list of 389 common contaminants (Supplementary file). The datasets presented in this software are ratios of the condition (e.g. after/before smoking a sham or real cigarette). The p-values were calculated from a standard Student’s t-test value, calculated as the average of the logs of the ratios of the individual peptides belonging to each protein divided by the standard error of the average of logs of the ratios of those peptides. Because of the large number of proteins considered in this experiment, p-values from the ProteinPilot analysis were adjusted to correct for multiple testing. In brief, for each of the four comparisons – 114:113, 116:115, 118:117, 121:119 – the Benjamini-Hochberg procedure was applied to convert the p-values to false discovery rate q-values using the *p.adjust*() function in R 3.6.3^31^.

We looked for an effect of smoking by comparing the after/before protein ratios for smokers and nonsmokers. Ratios significantly greater than 1 indicate a differential increase in a given protein after sham or cigarette smoking and ratios significantly lower than 1 indicate a differential decrease in a given protein after sham or cigarette smoking.

In addition to discovered proteins, we measured the following markers of stress.

### Salivary alpha amylase (sAA) protein expression by western blot

Equal amounts of (10 μg) saliva proteins were separated on 10% SDS-PAGE gels and transferred on polyvinylidene fluoride membrane. Anti-salivary alpha amylase and β-actin (Santa Cruz Biotechnology, Dallas, TX) antibodies were reacted with the blots at 1:1000 at 4^o^C. Following washings in TBS-Tween, horseradish peroxidase (HRP)-conjugated anti-goat secondary antibody was used at a dilution of 1:3000. Band expressions were developed using Pierce^TM^ ECL reagents (Thermo Scientific, Rockford, IL) and band densities were quantified using Image J analysis (National Institute of Health, Bethesda, MD). Fold change in band densities of sAA protein were normalized to band density of β-actin for all the samples.

### Salivary cortisol levels

Salivary cortisol, yet another established stress related marker^32,33^ was measured in saliva samples by commercially available ELISA kit (Salimetrics, State College, PA).

### Enzyme activity for sAA

The sAA activity was measured in duplicate in all the saliva samples from smokers and non-smokers following the instructions provided with Salimetrics Kit (Salimetrics, State College, PA).

### Salivary fibrinogen alpha and cystatin A levels

Salivary fibrinogen alpha and cystatin A were measured by commercial ELISA kits manufactured by Nova Lifetech Limited (Mongkok Ki, Hong Kong) and Assaypro LLC (St Charles, MO), respectively.

### Statistical analysis and power

Quantitative levels of fibrinogen alpha, cystatin A, sAA (western blot analysis) and salivary cortisol were recorded for both smokers (n=5) and nonsmokers (n=4) after both smoking sessions (cigarette smoking/sham smoking). SAS PROC MIXED (SAS 9.4, SAS Institute, Cary, NC, USA) was used to fit repeated measures linear models to compare protein levels between smokers and non-smokers. Models were fit using either smoking status alone or smoking status and one additional covariate (baseline age or weight). More complicated multivariable models were not used because of the small number of observations. Each protein difference was modeled separately, and all tests were performed at α=0.05 level. Additional analysis was performed to observe any association between the protein ratios in smokers and non-smokers after smoking (or sham smoking) Cig 1 and Cig 2 using Spearman’s correlation (r_s_). A previous proteomic profile of smokers using 5 subjects per group found significant differential expression of proteins, and was used for the sample size determination of the current study^28^.

## RESULTS

### iTRAQ analysis

A total of 484 proteins were identified in the pooled saliva samples by iTRAQ analysis (Supplementary file Table S1). The NCBI curated RefSeq protein sequence database, against which observed MS/MS spectra were searched by ProteinPilot (AB SCIEX, Framingham, MA), lists only one protein sequence per protein (to avoid duplicate entries). Thus, when a preproprotein version of secreted proteins is known, that is the single full-length sequence that is listed in the curated RefSeq protein sequence database, then many confident protein IDs are listed by name as the ‘preproprotein’ version of the sequence. However, manual inspection of the actual peptide sequences confidently identified shows that all of the peptides which were confidently identified and assigned to these full ‘preprotein’ sequences corresponded to peptides from the normally cleaved mature form of the identified proteins. With no peptides identified from the N-terminal cleaved pre-protein segments, the peptides which were identified are therefore consistent with assignment of the protein ID corresponding to the peptides identified as being the mature secreted forms of the proteins.

### Characteristics of proteins identified in iTRAQ from saliva of smokers and non-smokers after smoking or sham smoking

**Figure 1:**
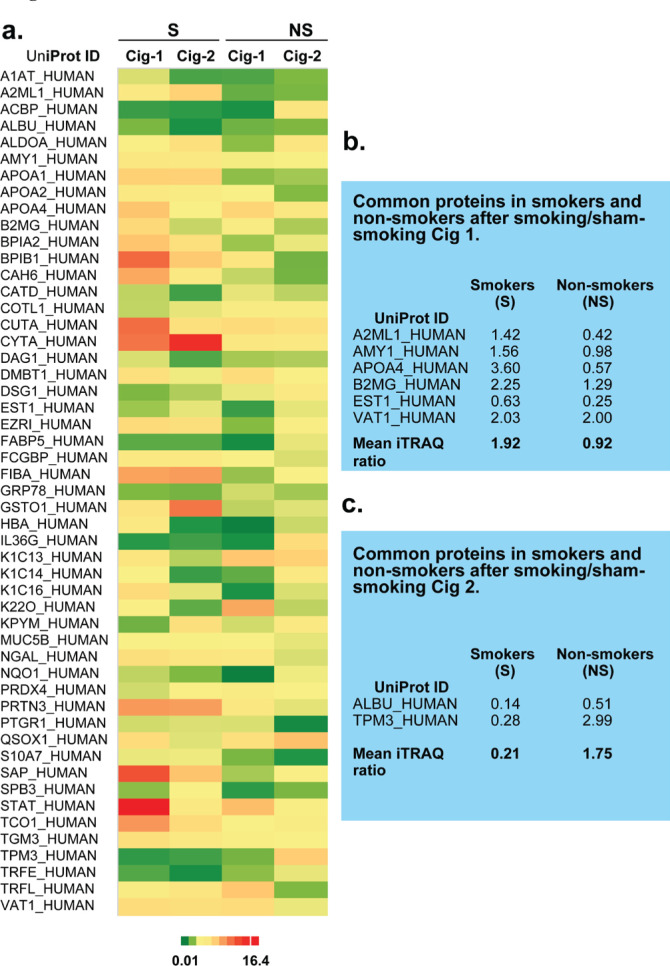
a) Heat map for differential salivary proteomics in smokers and non-smokers following smoking/sham smoking Cig 1 and Cig 2. b) List of common proteins expressed in saliva of smokers vs non-smokers after smoking/sham smoking Cig 1. c) List of common proteins expressed in saliva of smokers vs non-smokers after smoking/sham smoking Cig 2

A heat map for subset of significant proteins depicts the differential expression of salivary proteins that were altered after smoking or sham smoking Cig 1 and Cig 2 in smokers and non-smokers (Figure 1a). Out of these, 24 proteins were significantly (p<0.05) altered in smokers and non-smokers after smoking Cig 1 and sham smoking, respectively (Table 1) and only six proteins were commonly altered among these (Figure 1b). After smoking/sham smoking Cig 2, a total of 18 and 19 proteins were significantly altered in smokers and non-smokers, respectively (Table 1) and only two proteins were common among these (Figure 1c). The 8 common proteins were changing most likely because of smoking a cigarette rather than an act of smoking a cigarette and this needs to be verified in the future (Figure 1b and 1c).

**Table 1:** List of proteins significantly altered in saliva of smokers or non-smokers after smoking and sham smoking, respectively, following iTRAQ analysis. The ratios of after/before in smokers and non-smokers are depicted along with the p-values and number of peptides for Cig 1 and Cig 2. P-Values were also significant based on q values († q<0.05; †† q<0.001). Proteins highlighted in grey are higher in smokers after smoking Cig 1 and Cig 2 compared to that in non-smokers after sham smoking Cig 1 and Cig 2

*UniProt ID (HUMAN)*	*Name of protein*	*Peptides*	*ACig1/BCig1*	*Cig 1 S*	*ACig2/BCig2*	*Cig 2 S*	*AShCig1/BShCig1*	*Cig 1 NS*	*AShCig2/BShCig2*	*Cig 2 NS*	*Reported in smokers*	*References*
				*p*		*p*		*p*		*p*		
A1AT	Alpha-1-antitrypsin precursor	12	0.86	0.790	0.31	0.084	0.33	0.045	0.52	0.080	Yes	24
A2ML1	Alpha-2-macroglobulin-like protein 1 precursor	61	1.42	0.041	2.65	0.016	0.42	0.021	0.49	0.077	No	
ACBP	Acyl-CoA-binding protein isoform 5	9	0.25	0.004	0.21	0.005	0.13	0.194	1.66	0.795	No	
ALBU	Serum albumin preproprotein	228	0.50	0.092	0.14	0.001^†^	0.46	0.004	0.51	0.011	Yes	24
ALDOA	Fructose-bisphosphate aldolase A isoform 1	13	1.12	0.404	1.91	0.421	0.56	0.183	1.66	0.037	No	
AMY1	Alpha-amylase 1 precursor	1724	1.55	0.003	1.38	0.002	0.98	0.005	1.00	0.840	Yes	24
ANXA6	Annexin A6 isoform 1	3	0.49	0.393	3.80	0.204	24.43	0.033	0.47	0.322	No	
APOA1	Apolipoprotein A-I preproprotein	13	2.88	0.817	2.75	0.875	0.57	0.008	0.65	0.008	Yes	34
APOA2	Apolipoprotein A-II preproprotein	2	1.45	0.047	1.22	0.053	1.02	0.752	0.52	0.244	Yes	35
APOA4	Apolipoprotein A-IV precursor	4	3.60	0.006	1.05	0.011	2.51	0.006	1.58	0.277	Yes	19
B2MG	Beta-2-microglobulin precursor	5	2.25	0.003	0.79	0.129	1.29	0.004	0.69	0.107	Yes	36
BPIA2	BPI fold-containing family A member 2 precursor	88	3.66	0.017	1.85	0.389	0.62	0.995	0.94	0.320	Yes	24
BPIB1	BPI fold-containing family B member 1 precursor	19	9.55	0.026	3.25	0.347	1.64	0.575	0.47	0.333	Yes	24
CAH1	Carbonic anhydrase 1	3	20.32	0.033	1.37	0.823	0.07	0.094	0.77	0.497	Yes	37
CAH6	Carbonic anhydrase 6 isoform 1 precursor	117	5.30	0.014	1.53	0.182	0.77	0.912	0.47	0.020	Yes	24
CATD	Cathepsin D preproprotein	12	0.76	0.865	0.28	0.003	0.92	0.915	0.74	0.631	Yes	38
CATF	Cathepsin F precursor	1	1.82	0.366	1.38	0.552	1.19	0.722	87.90	0.019	No	
COPG2	Coatomer subunit gamma-2	1	0.48	0.314	0.27	0.184	0.01	0.063	87.90	0.017	No	
COTL1	Coactosin-like protein	3	0.77	0.338	0.92	0.006	1.25	0.272	1.24	0.314	Yes	24
CRYAA	Alpha-crystallin A chain	3	69.18	0.019	3.53	0.190	0.19	0.147	2.88	0.027	Yes	39
CUTA	Protein CutA isoform 1	3	9.20	0.213	1.82	0.421	2.13	0.413	1.92	0.045	No	
CYTA	Cystatin-A	13	8.63	0.004	13.93	0.002	1.57	0.066	1.51	0.912	Yes	24
DAG1	Dystroglycan preproprotein	5	0.86	0.836	0.34	0.031	0.67	0.669	0.70	0.411	No	
DMBT1	Deleted in malignant brain tumors 1 protein isoform c precursor	135	1.91	0.023	0.97	0.858	2.01	0.051	1.13	0.737	Yes	20
DSG1	Desmoglein-1 preproprotein	14	0.51	0.004	0.70	0.201	0.94	0.740	1.38	0.468	No	
EST1	Liver carboxylesterase 1 isoform a precursor	9	0.63	0.035	0.91	0.106	0.25	0.014	0.91	0.739	No	
EZRI	Ezrin	12	2.15	0.835	1.94	0.449	0.53	0.032	1.18	0.450	Yes	40
FABP5	Fatty acid-binding protein, epidermal	21	0.38	0.179	0.38	0.042	0.11	0.020	0.93	0.679	Yes	41
FCGBP	IgGFc-binding protein precursor	45	1.43	0.149	1.42	0.672	1.01	0.334	0.86	0.016	Yes	24
FIBA	Fibrinogen alpha chain isoform alpha preproprotein	2	5.75	0.029	6.19	0.014	0.60	0.478	1.01	0.960	Yes	34
GRP78	78 kDa glucose-regulated protein precursor	19	0.52	0.018	0.47	0.017	0.82	0.591	0.65	0.558	Yes	22
GSTO1	Glutathione S-transferase omega-1 isoform 1	2	1.77	0.420	8.55	0.033	0.75	0.732	0.89	0.692	Yes	42
HBA	Hemoglobin subunit alpha	8	1.53	0.503	0.18	0.397	0.02	0.014	0.79	0.376	Yes	43
IL36G	Interleukin-36 gamma	1	0.20	0.292	0.27	0.234	0.14	0.193	2.05	0.048	No	
K1C13	Keratin, type I cytoskeletal 13 isoform a	15	1.67	0.431	0.72	0.666	3.53	0.037	2.78	0.802	No	
K1C14	Keratin, type I cytoskeletal 14	16	1.04	0.153	0.25	0.184	0.41	0.011	1.47	0.447	No	
K1C16	Keratin, type I cytoskeletal 16	14	2.17	0.181	0.92	0.260	0.16	0.135	0.86	0.038	Yes	44
K22O	Keratin, type II cytoskeletal 2 oral	10	1.08	0.863	0.40	0.112	5.35	0.034	0.75	0.497	No	
KPYM	Pyruvate kinase isozymes M1/M2 isoform a	15	0.45	0.056	1.94	0.351	0.82	0.177	1.36	0.001	Yes	45
MUC5B	Mucin-5B precursor	309	1.01	0.145	1.00	0.166	1.02	0.045	0.91	0.005	Yes	25
NGAL	Neutrophil gelatinase-associated lipocalin precursor	15	1.94	0.021	1.60	0.599	1.58	0.136	0.85	0.889	Yes	46
NQO1	NAD(P)H dehydrogenase [quinone] 1 isoform b	1	0.76	0.621	0.52	0.344	0.01	0.046	0.97	0.965	Yes	47
OLFM4	Olfactomedin-4 precursor	4	0.64	0.490	99.08	0.017	0.33	0.229	0.60	0.433	No	
PRDX4	Peroxiredoxin-4 precursor	6	0.82	0.064	1.12	0.125	1.31	0.004	1.07	0.235	No	
PRTN3	Myeloblastin precursor	13	6.37	0.028	6.03	0.028	1.57	0.194	0.90	0.609	No	
PTGR1	Prostaglandin reductase 1 isoform 1	8	0.82	0.890	0.87	0.637	0.86	0.869	0.09	0.020	Yes	48
QSOX1	Sulfhydryl oxidase 1 isoform a precursor	4	2.09	0.724	0.89	0.236	1.80	0.094	3.77	0.028	No	
RAB5C	RAS-related protein Rab-5C isoform b	1	1.06	0.891	0.65	0.482	2.31	0.276	80.91	0.020	No	
S10A7	Protein S100-A7	22	0.94	0.099	0.96	0.361	0.49	0.026	0.18	0.003	No	
SAP	Prosaposin isoform a preproprotein	10	11.38	0.022	3.70	0.179	0.66	0.758	1.13	0.812	No	
SODE	Extracellular superoxide dismutase [Cu-Zn] precursor	1	2.11	0.306	3.34	0.198	0.36	0.232	46.13	0.020	Yes	21
SPB3	Serpin B3	29	0.56	0.633	1.05	0.096	0.22	0.004	0.50	0.064	Yes	23
STAT	Statherin isoform a precursor	133	16.44	0.002	1.51	0.409	3.94	0.084	1.17	0.442	No	
TCO1	Transcobalamin-1 precursor	20	6.61	0.029	2.17	0.135	1.05	0.845	1.22	0.219	Yes	49
TGM3	Protein-glutamine gammaglutamyltransferase E	19	1.98	0.007	1.53	0.825	1.16	0.481	1.07	0.531	No	
TPM3	Tropomyosin alpha-3 chain isoform 2	5	0.22	0.065	0.28	0.021	0.47	0.047	2.99	0.026	No	
TRFE	Serotransferrin precursor	61	0.35	0.088	0.13	0.001^††^	0.56	0.596	0.93	0.488	Yes	24
TRFL	Lactotransferrin isoform 1 preprotein	115	1.33	0.254	1.63	0.240	3.40	0.024	0.52	0.650	SHS	50
VAT1	Synaptic vesicle membrane protein VAT-1 homolog	5	2.03	0.003	1.96	0.003	2.00	0.003	0.94	0.731	Yes	24
ZG16B	Zymogen granule protein 16 homolog B precursor	63	19.95	0.081	23.12	0.107	2.00	0.011	0.81	0.072	Yes	51

Cig: cigarette. ACig: after smoking Cig. BCig: before smoking Cig. AShCig: after sham smoking Cig. BShCig: before sham smoking Cig. Cig 1: Cigarette-1. Cig 2: Cigarette-2. S: smokers. NS: non-smokers. SHS: secondhand smoke.

Table 1 describes the ratios of differentially expressed proteins identified in smokers and nonsmokers after smoking and sham smoking cigarettes, respectively. Several of these proteins were increased in smokers after smoking Cig 1 and Cig 2. To observe similarities in protein fold changes before and after Cig 1 and Cig 2 in smokers and non-smokers, a Spearman’s correlation was also performed. Data analysis on 60 proteins from smokers showed r_s_=0.62369, p(2-tailed)=1.019×10^-7^, and from nonsmokers showed r_s_=0.03221 , p(2-tailed )=0.80697.

### Validating iTRAQ data

**Figure 2:**
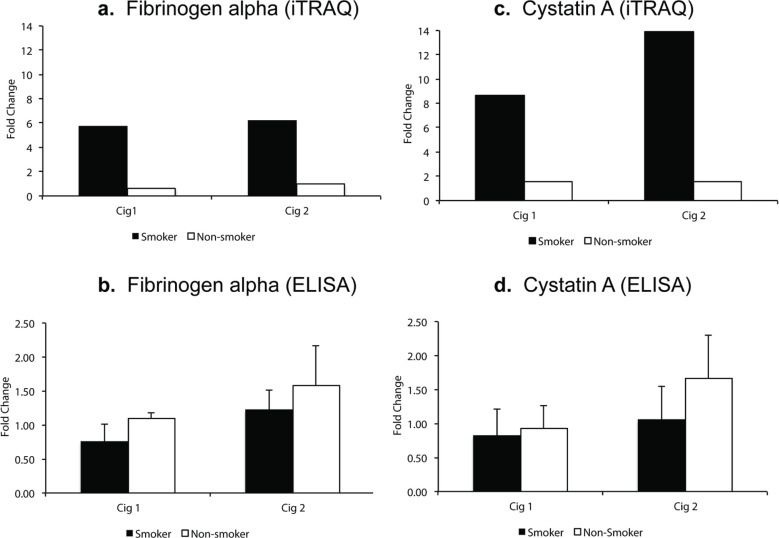
Salivary fibrinogen alpha and cystatin A levels following iTRAQ analysis and validation by ELISA in smokers vs non-smokers. Fold change indicates ratio of protein levels after/before smoking/sham smoking in smokers and non-smokers, respectively. a) fibrinogen alpha levels from iTRAQ analysis. b) fibrinogen alpha levels by ELISA. c) cystatin A levels from iTRAQ analysis. d) cystatin A levels by ELISA

For validating iTRAQ data, initially, from the list of proteins that showed a high fold-change, were found to be significant (ProteinPilot p-value) in both smoking sessions for smokers (Table 1). Next, we selected proteins fibrinogen alpha and cystatin A for which ELISA kits were available and also included a protein (sAA) that was in fact *a priori* hypothesized protein associated with stress response based on a review of the literature as well as it had the maximum peptide hits for protein identification. In iTRAQ analysis, fibrinogen alpha was significantly elevated 5.8-fold and 6.2-fold (p<0.05, q>0.05) in smokers after smoking Cig 1 and Cig 2, respectively. In non-smokers, however, it was reduced 0.6-fold and unchanged (p>0.05, q>0.05) after sham smoking Cig 1 and Cig 2, respectively (Figure 2a). Levels of fibrinogen alpha measured in individual saliva samples by Commercial ELISA revealed a 0.8-fold reduction and 1.2-fold increase (p>0.05) in fibrinogen levels in smokers after smoking Cig 1 and Cig 2, respectively. While in non-smokers fibrinogen levels were non-significantly elevated 1.1-fold and 1.58-fold after sham smoking Cig 1 and Cig 2, respectively (Figure 2b). Interestingly, when smoking status and weight of participants were considered in a statistical model, the fibrinogen alpha levels measured by ELISA were significantly different (p<0.05) among smokers and non-smokers after both smoking sessions (Supplementary file Table S2).

In iTRAQ analysis of pooled samples, cystatin A level was significantly increased 8.6-fold and 13.9-fold (p<0.005, q>0.05) in smokers after smoking Cig 1 and Cig 2, respectively, and in non-smokers it was elevated 1.5-fold (p>0.05, q>0.05) after sham smoking Cig 1 and Cig 2 (Figure 2c). Upon analyzing the individual smoker and non-smoker saliva samples by Commercial ELISA kit, cystatin A levels were increased 1.65-fold (p>0.05) in non-smokers after sham smoking Cig 2, while in smokers no significant changes were observed (Figure 2d). The fold changes in iTRAQ analysis could not be validated in ELISA and one of the reasons for this difference may be pooling the samples for the former method. Additionally, when smoking status and age or weight of participants were considered in a statistical model, the cystatin A levels measured by ELISA were not statistically different (p>0.05) among smokers and non-smokers after both smoking sessions (Supplementary file Table S2).

**Figure 3:**
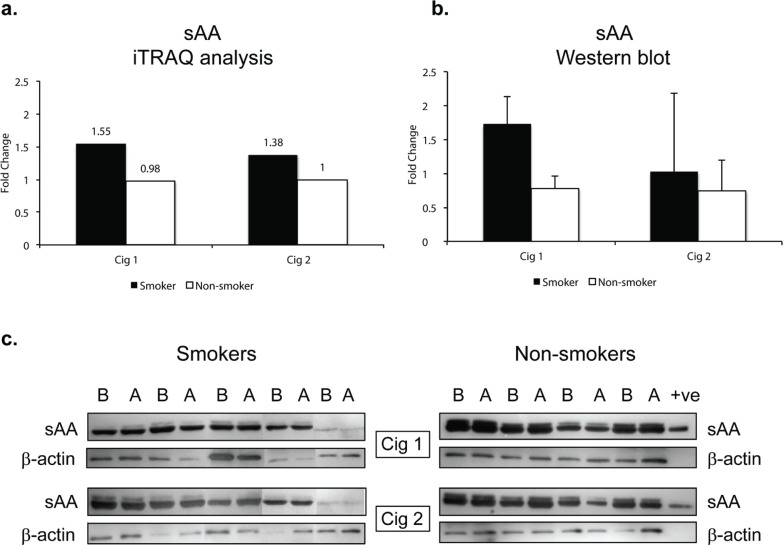
Salivary alpha amylase (sAA) relative levels in smokers and non-smokers. a) sAA levels following iTRAQ analysis. b) sAA levels measured by western blot analysis. The sAA bands were normalized with corresponding β-actin levels for each sample. c) western blots for sAA in smokers and non-smokers. Fold changes in a) and b) indicate ratios of sAA levels after/before smoking/sham smoking in smokers and non-smokers, respectively. B before and A after smoking (or sham smoking in non-smokers). N=5 (smokers), N=4 (non-smokers).

sAA was significantly elevated after smoking Cig 1 and Cig 2 in smokers by 1.6-fold and 1.4-fold (p<0.005, q>0.05), respectively, as determined by iTRAQ data analysis (Figure 3a, Table 1). However, in non-smokers, sAA did not change after sham smoking Cig 1 and Cig 2.

Since sAA has been reported to be a marker for stress we wanted to examine if there were any modifications in sAA in the saliva. The iTRAQ analysis was based on pooled samples so we additionally examined individual saliva specimens by western blotting. Figure 3b depicts the quantitative data as a ratio of ‘after/before’ smoking Cig 1 and Cig 2 in smokers as well as ‘after/before’ sham smoking Cig 1 and Cig 2 in non-smokers. β-actin was used to normalize the data presented in fold change of sAA in smokers versus non-smokers. Figure 3c shows the sAA bands reacting with anti-sAA antibody in western blot analysis. Overall, baseline sAA protein bands showed higher intensity in non-smokers compared to smokers but the fold change after smoking was greater in smokers than non-smokers, yet not significant. An additional band running just above sAA likely being the glycosylated form of sAA was also expressed at higher intensity in non-smokers compared to smokers. When smoking status and age or weight of participants were considered in a statistical model, sAA levels measured by western blot showed a trend but were not statistically different (p=0.085) among smokers and non-smokers after both the smoking sessions (Supplementary file Table S2).

### Impact of smoking/sham-smoking on salivary cortisol

The salivary cortisol levels were increased after smoking as well as sham smoking Cig 2 in smokers and non-smokers, respectively; however, there was a decrease in cortisol after Cig 1 in smokers as well as in non-smokers (Table 2). When smoking status and age or weight of participants were considered in a statistical model, the salivary cortisol levels were not statistically different (p>0.05) among smokers and non-smokers after both smoking sessions (Supplementary file Table S2).

**Table 2:** Comparative sAA enzyme activity and cortisol levels in saliva of smokers and non-smokers before and after smoking/sham smoking Cig 1 and Cig 2

*Enzyme activity*	*Before Cig 1 Mean ± SE*	*After Cig 1 Mean ± SE*	*Before Cig 2 Mean ± SE*	*After Cig 2 Mean ± SE*
Smokers sAA enzyme activity (U/mL)	35.75 ± 11.83	33.59 ±11.26	32.14 ± 10.95	33.85 ± 10.42
Non-smokers sAA enzyme activity (U/mL)	59.45 ± 15.20	62.73 ± 18.52	61.91 ± 14.33	71.50 ± 22.0
Smokers Cortisol (μg/dL)	0.13 ± 0.03	0.10 ± 0.03	0.08 ± 0.03	0.10 ± 0.03
Non-smokers Cortisol (μg/dL)	0.20 ± 0.02	0.17 ± 0.01	0.14 ± 0.01	0.15 ± 0.02

## DISCUSSION

This study sought to determine the feasibility of using proteomics in human subjects’ research to identify new protein markers in the saliva that change in response to an acute smoking event versus acute sham smoking event in smokers and non-smokers, respectively. Utilizing iTRAQ methodology we were able to identify proteins that might assist in understanding changes that occur in the stress response to smoking. This new information might assist in improving methods for behavioral and pharmacological interventions for smokers. A total of 484 salivary proteins were identified by iTRAQ method in our pilot study as being affected by cigarette smoking. Some of these proteins were reported for the first time in saliva of smokers.

Some of the proteins identified in the saliva of smokers after smoking a cigarette in our study have previously been reported in biomarker studies of smokers^19-25,33-51^ and several new proteins were reported for the first time in saliva of smokers in our analysis.

Furthermore, protein profiling revealed that the common proteins amongst smokers increased about 2-fold on average (Figure 1b), while these were unchanged in non-smokers, indicating that after Cig 1 these protein changes were indeed impacted by smoking. Based on the proteins identified in our pilot study, we can begin to categorize proteins that change as a result of smoking and that have a role in nicotine dependence in smokers: AMY1, CAH1, CAH6, BPIA2, BPIB1, TCO1, NGAL, GSTO1 A2ML1, TGM3, and SAP are potential candidate proteins that are recommended to validate in a larger smoker study.

Several studies have shown that psychosocial and physical stressors can rapidly increase the sAA activity. Our study showed changes in sAA within 30 min of smoking a cigarette. In fact, sAA has been proposed as a potential non-invasive biomarker for SNS^52^. Salivary amylase along with glutathione S-transferase, and prolactin-induced protein have been shown to correlate with psychosocial stress^4^. In another study, acute stress increased salivary amylase and cystatins in medical students exposed to clinical simulations^5^. Genetically, sAA is coded by AMY1 gene and its copy number is positively correlated with sAA protein expression in humans, and that sAA amount is also positively correlated with its activity^53,54^. About 40–50% of saliva is composed of sAA^52^ and the latter is a mixture of two isoenzymes as glycosylated and non-glycosylated that differ by just 4 kDa. However, it is still not clear if these two isoenzymes differ in their clinical roles. In our western blot analysis, we noted both glycosylated and non-glycosylated forms of sAA in saliva of non-smokers as well as smokers (Figure 3c). In a future study we will investigate the role of glycosylated sAA in behavior of smokers.

sAA activity is conventionally measured in saliva samples for studies analyzing psychosocial stress but, examining sAA activity in smokers may pose a potential technical problem. The aldehydes in smoke diminish the sAA activity in saliva^55^, therefore it may be underestimating the sAA activity in saliva samples from smokers.

Among other proteins that exhibited a smokingresponse, GSTO1, GRP78, and APOA4 have been reported to be involved in psychological stress^4^, mood disorders^56^, and depression^57^, respectively.

Our study employed a novel arm of sham smoking in non-smokers. Interestingly, we found protein changes in this group. It may be the act of smoking rather than nicotine exposure is associated with an acute response to changes in stress. Future studies may need to consider control conditions such as these to further understand whether the proteomic response to smoking is due to smoking behaviors or to nicotine exposure.

### Strengths and limitations

The iTRAQ analysis of smokers (n=5) and non-smokers (n=4) in our pilot study identified numerous proteins in saliva for the first time. There were consistent changes in proteins in repeating the smoking as well as sham-smoking sessions in smokers and non-smokers, respectively. Evidently, there were important lessons learned while analyzing these samples. For an initial approach using iTRAQ, pooling of samples was opted to obtain changes in global proteomic profile but, when we investigated these changes in individual samples by ELISA, the overall fold-change for selected proteins was not similar as noted for cystatin A and was true for fibrinogen. In western blot analysis for sAA, however, the trend was similar as observed in iTRAQ for smokers after smoking Cig 1. It will be critical to label individual saliva samples with a unique tag in order to measure an appropriate quantitative change rather than pooling the samples from each group. By pooling the samples, the assessment of potential group differences was potentially weakened. In addition, it is imperative to validate the results of iTRAQ by one or more methods specifically, if samples have been pooled initially. Findings are not entirely in the direction we expected. For example, cortisol levels were higher in non-smokers compared to smokers in this study although the literature also is inconsistent in this area. However, preliminary findings from our small study, with interesting behavior-related protein markers, could be replicated in a larger set of saliva samples from smokers and non-smokers in the future.

## CONCLUSIONS

Our iTRAQ analysis identified several new proteins in saliva of smokers. Some of these novel proteins may respond to physiological or psychological stressors in smokers following smoking. However, further studies are needed to validate these findings and future experiments will require their validation in a larger sample size. Moreover, an understanding of combined expression for some of these proteins in addition to sAA and cortisol levels could potentially lead to new and improved methods for behavioral and pharmacological interventions. Candidate proteins that would be interesting to validate include: ACBP, A2ML1, APOA4, BPIB1, BPIA2, CAH1, CAH6, DSG1, EST1, GRP78, GSTO1, sAA, SAP, STAT, TCO1, and TGM3, in addition to cortisol.

## Data Availability

The data supporting this research can be found in the Supplementary file.
