# Supplementary material for: Changes in salivary proteome before and after cigarette smoking in smokers compared to sham smoking in nonsmokers: A pilot study
Source: Tob Induc Dis. 2021 Jun 29;19:56. doi: 10.18332/tid/138336 (PMC8240953; doi:10.18332/tid/138336)
Supplement: Supplementary file 1 [file TID-19-56-s1.pdf]

Details on experimental procedures involving iTRAQ analysis.

2D-LC Separations

First dimension separation of the peptides of the 8-plex iTRAQ reagent labeled samples was accomplished by strong cation exchange chromatography (SCX) using a PolySULFOETHYL Aspartide column (4.6 x 250 mm, PolyLC, Columbia, MD) on a Waters 600E HPLC System using 20% acetonitrile in 10 mM aqueous ammonium formate, pH 2.7 (buffer A), and 20% acetonitrile in 666 mM aqueous ammonium formate, pH 2.7 (buffer B). The flow rate was 1 ml/min and the elution program was 100% A for 22 min, 0% to 40% B from 22 to 48 min, 40% to 100% B from 48 to 49 min, hold at 100% B to for 7 min before returning to 100% A. The first 28 ml were collected as fraction 1, followed by collection of fractions 2-15, 2 ml each. All fractions were evaporated to dryness, then resuspended in 9 µl of aqueous 2% acetonitrile (v/v) and 0.1% trifluoroacetic acid (TFA, v/v) and filtered prior to reverse phase C18 nanoflow-LC separation.

Second dimension separation of the collected SCX fractions was accomplished by reverse phase chromatography using a Chromolith CapRod column (150 x 0.1 mm, Merck, Gibbstown, NJ) on a Tempo LC Matrix-Assisted Laser Desorption/Ionization (MALDI) Spotting system (ABI-MDS/Sciex). The injector loop was 5 µl. Buffer C was 2% acetonitrile, 0.1% TFA in water and Buffer D was 98% acetonitrile, 0.1% TFA. The elution program was 95%C/5%D at 2 µl/min from 0-3 min, an increase in flow rate to 2.5 µl/min from 3 to 8.1 min, then 5% D to 38% D from 8.1 to 40 min, 38%D to 80% D from 41 to 44 min, followed by a return to initial conditions (5% D) from 44 to 49 min. The MALDI matrix solution, consisting of 7 mg/ml of recrystallized α-cyano-hydroxycinnamic acid, 2 mg/ml ammonium phosphate, in 80% acetonitrile with 0.1% TFA, was added at a rate of 2.5 µl/min post-column to the HPLC eluent. The combined solution was automatically spotted (0.5 µl/spot) onto a stainless steel MALDI target plate every 6 seconds for a total of 370 spots per original fraction obtained from the first dimension SCX separation.

Mass Spectrometric (MS) Analysis

After sample spot drying, 13 calibrant spots (ABI 4700 Mix) were added to each plate manually. MALDI target plates (15 per experiment) were analyzed in a data-dependent manner on an ABI 5800 MALDI TOF/TOF. As each plate entered into the instrument, a plate calibration/ MS Default calibration update was performed, and then the MS/MS default calibration was updated. MS Spectra were taken from 5,500 MALDI Spots, using 500 laser shots per spot at laser power 2600. A plate-wide interpretation was then automatically performed, choosing the highest peak of each observed m/z value for subsequent MS/MS analysis. Up to 2500 laser shots at laser power 3200 with collision-induced dissociation gas Air at 1.2 to 1.3 x 10^{-6} Torr were accumulated for each MS/MS spectrum taken (total: 15,492 MS/MS spectra).

Database Search for Protein Identification and Quantitation

Protein Pilot™ search parameters were set at: Cys Alkylation – Iodoacetamide; ID Focus – Biological Modifications; Search Effort – Thorough. MS and MS/MS spectra were searched using the Human NCBI database sequences containing 513,785 protein sequences, plus 389 common lab contaminants. Total protein sequences searched in database plus contaminants plus 1,025,884 concatenated reverse decoy database. The Local or "Instantaneous" FDR estimate combined with the Pro Group™ Algorithm included in Protein Pilot™ gives a very conservative and fully Minimum Information About a Proteomics Experiment (MIAPE)-compliant list of proteins identified. The unused protein score is a measure of the protein confidence for a detected protein, calculated from the peptide confidence for peptides from spectra that are not already completely "used" by higher scoring winning proteins (Protein Pilot™ 4.5). The Local FDR is estimates the "local" error rate around a given identification, which indicates the likelihood that that the specific identification is incorrect based on the use of Decoy Database searches (either Reversed or Randomized version of the same Forward/Normal database used for searching), presumably containing no real sequences, with the assumption that the number of IDs of Decoy (not real) peptides or proteins at a particular threshold accurately estimates the number of FALSE identifications from the Forward/Normal database.

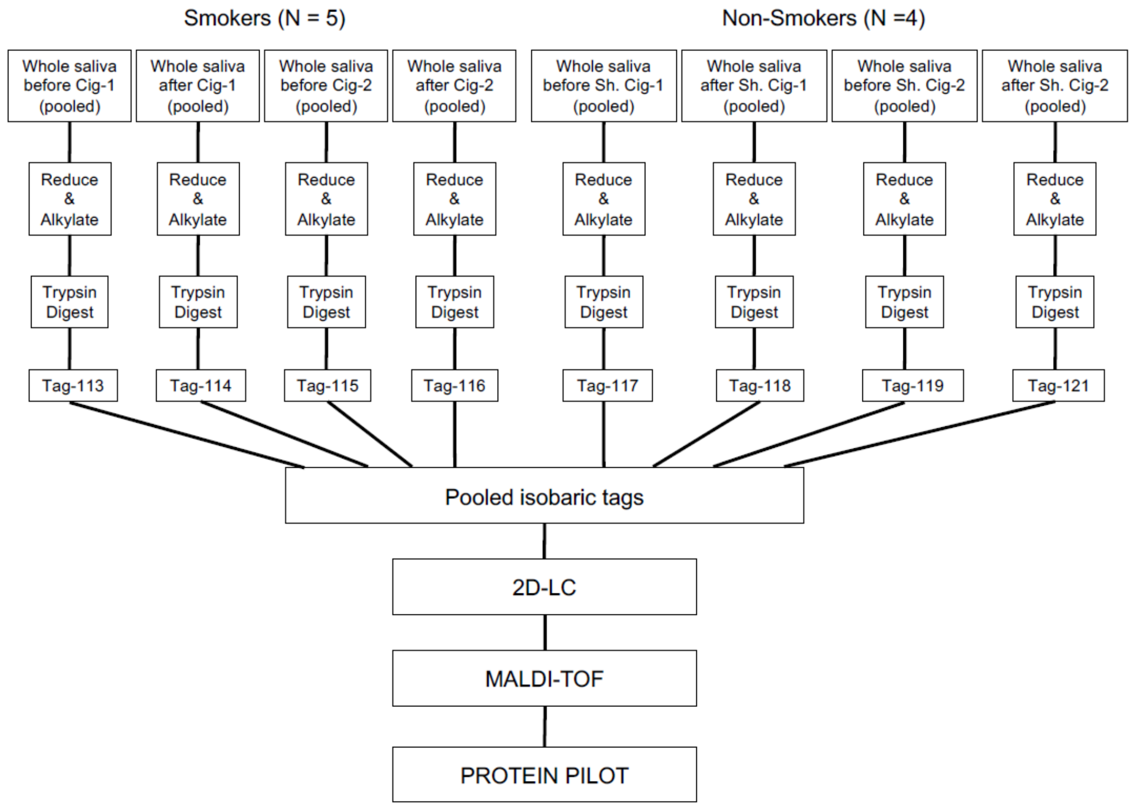

Supplementary figure S1

Table S1. Description of proteins identified by iTRAQ in saliva of smokers and non-smokers before and after smoking or sham smoking respectively.

The ratios of after/before in smokers and non-smokers are depicted along with the P-Values and number of peptides for Cig 1 and Cig 2.

Accession #	Uniprot ID	Protein Name	Peptides (95%)	Ratio ACig1/BCig1	P-Value Cig1 S	Ratio ACig2/BCig2	P-Value Cig2 S	Ratio AShCig1/BShCig1	P-Value Cig1 NS	Ratio AShCig2/BShCig2	P-Value Cig2 NS
gi|189458812	GLGB_HUMAN	1,4-alpha-glucan-branching enzyme	1	0.10	0.606	2.68	0.452	1.08	0.556	0.21	0.368
gi|5454052	1433S_HUMAN	14-3-3 protein sigma	17	0.54	0.920	0.29	0.745	0.52	0.190	0.24	0.450
gi|4507953	1433Z_HUMAN	14-3-3 protein zeta/delta	23	0.73	0.606	0.29	0.079	0.77	0.621	1.14	0.188
gi|14277700	RS12_HUMAN	40S ribosomal protein S12	1	1.89	0.349	1.27	0.635	0.09	0.102	0.54	0.367
gi|9845502	RSSA_HUMAN	40S ribosomal protein SA	1	1.09	0.738	0.59	0.320	0.67	0.513	0.34	0.481
gi|7706573	CAB45_HUMAN	45 kDa calcium-binding protein isoform 1 precursor	1	1.11	0.823	1.21	0.693	1.14	0.790	0.74	0.602
gi|40068518	6PGD_HUMAN	6-phosphogluconate dehydrogenase, decarboxylating	16	1.03	0.632	0.93	0.766	1.01	0.195	0.83	0.762
gi|6912586	6PGL_HUMAN	6-phosphogluconolactonase	2	0.46	0.455	6.43	0.290	1.04	0.962	2.23	0.732
gi|31542947	CH60_HUMAN	60 kDa heat shock protein, mitochondrial	1	1.34	0.615	0.86	0.863	1.91	0.330	1.47	0.493
gi|4506667	RLA0_HUMAN	60S acidic ribosomal protein P0	2	1.06	0.779	0.90	0.665	0.61	0.152	1.06	0.778
gi|4506669	RLA1_HUMAN	60S acidic ribosomal protein P1 isoform 1	1	0.10	0.438	0.35	0.369	9.38	0.426	0.08	0.231
gi|4506671	RLA2_HUMAN	60S acidic ribosomal protein P2	3	0.74	0.660	1.87	0.712	1.50	0.473	0.13	0.173
gi|16507237	GRP78_HUMAN	78 kDa glucose-regulated protein precursor	19	0.52	0.018	0.47	0.017	0.82	0.591	0.65	0.558
gi|148539872	THIC_HUMAN	Acetyl-CoA acetyltransferase, cytosolic	1	0.42	0.270	0.45	0.293	0.47	0.310	1.43	0.518
gi|189011550	ASAH1_HUMAN	Acid ceramidase isoform c	1	1.67	0.407	1.42	0.530	4.83	0.154	0.56	0.382
gi|5453880	AN32A_HUMAN	Acidic leucine-rich nuclear phosphoprotein 32 family member A	1	0.63	0.458	0.94	0.916	1.16	0.759	1.61	0.429
gi|53692187	ARP2_HUMAN	Actin-related protein 2 isoform a	2	1.19	0.700	1.20	0.710	1.74	0.386	1.21	0.701
gi|5031599	ARPC2_HUMAN	Actin-related protein 2/3 complex subunit 2	1	4.17	0.281	2.83	0.344	0.95	0.958	0.03	0.175

gi|5031595	ARPC4_HUMAN	Actin-related protein 2/3 complex subunit 4 isoform a	2	2.47	0.528	0.51	0.122	0.83	0.910	0.57	0.147
gi|5031593	ARPC5_HUMAN	Actin-related protein 2/3 complex subunit 5 isoform 1	1	2.73	0.235	0.84	0.745	1.02	0.954	1.26	0.654
gi|5031573	ARP3_HUMAN	Actin-related protein 3	3	1.60	0.849	3.25	0.134	0.90	0.867	0.89	0.519
gi|4501887	ACTG_HUMAN	Actin, cytoplasmic 2	84	0.84	0.133	1.57	0.326	0.97	0.547	1.46	0.562
gi|295842514	ACBP_HUMAN	Acyl-CoA-binding protein isoform 5	9	0.25	0.004	0.21	0.005	0.13	0.194	1.66	0.795
gi|32484975	ADK_HUMAN	Adenosine kinase isoform b	1	1.33	0.590	0.90	0.843	0.83	0.727	2.47	0.258
gi|9951915	SAHH_HUMAN	Adenosylhomocysteinase isoform 1	1	1.50	0.479	0.92	0.881	1.39	0.546	0.63	0.457
gi|5453595	CAP1_HUMAN	Adenylyl cyclase-associated protein 1	7	0.51	0.053	1.57	0.165	1.38	0.250	1.74	0.191
gi|5802976	ADIRF_HUMAN	Adipose most abundant gene transcript 2 protein	1	0.74	0.481	1.43	0.563	0.75	0.889	0.46	0.399
gi|4502203	ARF3_HUMAN	ADP-ribosylation factor 3	1	0.70	0.538	0.55	0.378	0.74	0.602	0.36	0.233
gi|5174391	AK1A1_HUMAN	Alcohol dehydrogenase [NADP(+)]	2	0.83	0.832	1.42	0.583	1.38	0.485	1.10	0.759
gi|262073058	ADH7_HUMAN	Alcohol dehydrogenase class 4 mu/sigma chain isoform 1	4	0.90	0.777	0.51	0.186	1.09	0.676	0.64	0.411
gi|22907049	AL3A1_HUMAN	Aldehyde dehydrogenase, dimeric NADP-preferring	4	0.83	0.326	1.85	0.711	1.77	0.770	1.27	0.847
gi|223468663	AK1BA_HUMAN	Aldo-keto reductase family 1 member B10	5	0.76	0.750	1.34	0.714	0.63	0.608	1.01	0.584
gi|167857790	A1AG1_HUMAN	Alpha-1-acid glycoprotein 1 precursor	6	0.68	0.876	0.29	0.485	0.85	0.765	1.53	0.457
gi|50659080	AACT_HUMAN	Alpha-1-antichymotrypsin precursor	5	2.54	0.777	2.54	0.900	0.75	0.905	0.35	0.617
gi|50363217	A1AT_HUMAN	Alpha-1-antitrypsin precursor	12	0.86	0.790	0.31	0.084	0.33	0.045	0.52	0.080
gi|21071030	A1BG_HUMAN	Alpha-1B-glycoprotein precursor	7	0.84	0.633	0.78	0.393	0.40	0.081	1.17	0.162
gi|156523970	FETUA_HUMAN	Alpha-2-HS-glycoprotein preproprotein	8	1.01	0.956	0.33	0.366	0.70	0.920	0.78	0.411
gi|66932947	A2MG_HUMAN	Alpha-2-macroglobulin precursor	41	0.99	0.236	1.50	0.965	0.88	0.479	0.85	0.980
gi|74271845	A2ML1_HUMAN	Alpha-2-macroglobulin-like protein 1 precursor	61	1.42	0.041	2.65	0.016	0.42	0.021	0.49	0.077
gi|194097350	ACTN1_HUMAN	Alpha-actinin-1 isoform a	14	1.67	0.780	3.44	0.079	0.66	0.449	1.75	0.432
gi|12025678	ACTN4_HUMAN	Alpha-actinin-4	16	0.80	0.813	0.60	0.933	0.39	0.301	1.98	0.466
gi|40254482	AMY1_HUMAN	Alpha-amylase 1 precursor	1724	1.56	0.003	1.38	0.002	0.98	0.005	1.00	0.840
gi|4503055	CRYAA_HUMAN	Alpha-crystallin A chain	3	69.18	0.019	3.53	0.190	0.19	0.147	2.88	0.027
gi|4503057	CRYAB_HUMAN	Alpha-crystallin B chain	2	1.31	0.608	10.47	0.105	0.33	0.216	0.19	0.148

gi|4503571	ENOA_HUMAN	Alpha-enolase isoform 1	85	0.67	0.739	0.74	0.106	0.65	0.146	0.82	0.118
gi|66346698	ANAG_HUMAN	Alpha-N-acetylglucosaminidase precursor	2	0.63	0.454	1.96	0.331	0.87	0.800	1.21	0.701
gi|40316915	AMPB_HUMAN	Aminopeptidase B	3	1.20	0.498	1.24	0.296	1.46	0.124	0.95	0.985
gi|4502167	A4_HUMAN	Amyloid beta A4 protein isoform a precursor	1	0.28	0.189	0.33	0.216	1.66	0.414	0.56	0.381
gi|4557287	ANGT_HUMAN	Angiotensinogen preproprotein	3	1.47	0.553	0.79	0.731	1.18	0.694	0.61	0.520
gi|4502101	ANXA1_HUMAN	Annexin A1	6	1.84	0.495	1.74	0.604	1.91	0.387	3.56	0.124
gi|4557317	ANX11_HUMAN	Annexin A11	1	0.61	0.434	0.50	0.331	2.70	0.236	0.98	0.981
gi|50845388	ANXA2_HUMAN	Annexin A2 isoform 1	3	0.92	0.879	0.30	0.200	1.85	0.356	1.31	0.606
gi|4502107	ANXA5_HUMAN	Annexin A5	2	0.45	0.148	1.54	0.375	0.83	0.654	1.16	0.613
gi|71773329	ANXA6_HUMAN	Annexin A6 isoform 1	3	0.49	0.393	3.80	0.204	24.43	0.033	0.47	0.322
gi|4502261	ANT3_HUMAN	Antithrombin-III precursor	1	4.49	0.460	1.67	0.238	0.73	0.760	0.43	0.337
gi|4557321	APOA1_HUMAN	Apolipoprotein A-I preproprotein	13	2.88	0.817	2.75	0.875	0.57	0.008	0.65	0.008
gi|4502149	APOA2_HUMAN	Apolipoprotein A-II preproprotein	2	1.45	0.047	1.22	0.053	1.02	0.752	0.52	0.244
gi|71773110	APOA4_HUMAN	Apolipoprotein A-IV precursor	4	3.60	0.006	1.05	0.011	2.51	0.006	1.58	0.277
gi|105990532	APOB_HUMAN	Apolipoprotein B-100 precursor	10	0.89	0.467	0.77	0.540	1.29	0.470	0.48	0.300
gi|22035620	ASC_HUMAN	Apoptosis-associated speck-like protein containing a CARD isoform b	1	0.66	0.490	1.67	0.407	1.07	0.878	0.72	0.557
gi|346986435	ARGI1_HUMAN	Arginase-1 isoform 1	3	1.45	0.409	0.95	0.668	0.86	0.806	1.13	0.626
gi|4504067	AATC_HUMAN	Aspartate aminotransferase, cytoplasmic	3	0.72	0.782	2.23	0.350	0.28	0.150	1.42	0.435
gi|38569423	ACLY_HUMAN	ATP-citrate synthase isoform 2	2	0.98	0.986	0.45	0.186	2.15	0.192	2.73	0.152
gi|21450863	ATRN_HUMAN	Attractin isoform 2 preproprotein	1	3.28	0.202	0.79	0.671	0.72	0.565	1.14	0.785
gi|11342670	CAP7_HUMAN	Azurocidin preproprotein	3	0.69	0.646	0.19	0.377	1.56	0.591	0.53	0.397
gi|157276599	BPI_HUMAN	Bactericidal permeability-increasing protein precursor	3	0.70	0.416	0.15	0.085	0.63	0.452	1.00	0.966
gi|4502389	BAF_HUMAN	Barrier-to-autointegration factor	1	2.29	0.274	2.38	0.286	0.70	0.518	0.79	0.627
gi|153266841	APOH_HUMAN	Beta-2-glycoprotein 1 precursor	6	1.47	0.476	0.35	0.380	0.72	0.904	1.25	0.877
gi|4757826	B2MG_HUMAN	Beta-2-microglobulin precursor	5	2.25	0.003	0.79	0.129	1.29	0.004	0.69	0.107
gi|9910390	CNBP1_HUMAN	Beta-catenin-interacting protein 1	1	0.56	0.414	0.39	0.265	0.72	0.615	1.28	0.613

gi|189181666	HEXA_HUMAN	Beta-hexosaminidase subunit alpha preproprotein	2	1.09	0.856	0.76	0.623	1.28	0.629	2.49	0.255
gi|84798622	MANBA_HUMAN	Beta-mannosidase precursor [Homo sapiens]	4	1.26	0.698	7.18	0.307	0.72	0.713	0.42	0.213
gi|20127454	PUR9_HUMAN	Bifunctional purine biosynthesis protein PURH	1	0.87	0.792	0.35	0.230	0.81	0.693	1.06	0.896
gi|7706119	BPIA1_HUMAN	BPI fold-containing family A member 1 precursor	6	2.81	0.111	0.70	0.284	1.29	0.905	0.83	0.157
gi|45592961	BPIA2_HUMAN	BPI fold-containing family A member 2 precursor	88	3.66	0.017	1.85	0.389	0.62	0.995	0.94	0.320
gi|40807482	BPIB1_HUMAN	BPI fold-containing family B member 1 precursor	19	9.55	0.026	3.25	0.347	1.64	0.575	0.47	0.333
gi|15055535	BPIB2_HUMAN	BPI fold-containing family B member 2 precursor	61	1.29	0.142	0.80	0.404	0.74	0.644	0.68	0.530
gi|4502503	C4BPA_HUMAN	C4b-binding protein alpha chain precursor	1	0.86	0.784	0.56	0.389	0.82	0.707	0.80	0.686
gi|4757960	CADH1_HUMAN	Cadherin-1 preproprotein	5	1.14	0.833	1.50	0.744	0.46	0.995	0.80	0.957
gi|163644313	CIB1_HUMAN	Calcium and integrin-binding protein 1	1	1.11	0.822	0.29	0.198	0.44	0.288	0.65	0.476
gi|150036262	CLCA4_HUMAN	Calcium-activated chloride channel regulator 4 precursor	1	1.09	0.859	0.54	0.458	1.24	0.721	0.28	0.273
gi|7706481	CAB39_HUMAN	Calcium-binding protein 39	2	0.59	0.413	1.50	0.476	0.84	0.747	0.61	0.424
gi|209364621	KCC1B_HUMAN	Calcium/calmodulin-dependent protein kinase type 1B isoform b	1	1.19	0.723	1.01	0.963	3.10	0.211	0.47	0.306
gi|4502549	CALM_HUMAN	Calmodulin	2	2.63	0.217	2.36	0.219	2.54	0.284	0.84	0.979
gi|223278387	CALL5_HUMAN	Calmodulin-like protein 5	2	1.24	0.948	1.27	0.691	0.33	0.596	0.70	0.911
gi|4502565	CPNS1_HUMAN	Calpain small subunit 1	1	0.47	0.305	1.14	0.781	0.45	0.296	1.84	0.359
gi|12408656	CAN1_HUMAN	Calpain-1 catalytic subunit	1	0.69	0.526	1.10	0.960	0.78	0.665	1.53	0.482
gi|157389005	CAN2_HUMAN	Calpain-2 catalytic subunit isoform 1	1	1.92	0.341	0.69	0.531	1.20	0.713	2.11	0.305
gi|4757900	CALR_HUMAN	Calreticulin precursor	3	1.16	0.698	0.60	0.352	1.06	0.845	1.04	0.932
gi|4502551	CALU_HUMAN	Calumenin isoform a precursor	1	0.82	0.719	0.80	0.682	0.93	0.896	1.33	0.592
gi|4502517	CAH1_HUMAN	Carbonic anhydrase 1	3	20.32	0.033	1.37	0.823	0.07	0.094	0.77	0.497
gi|4557395	CAH2_HUMAN	Carbonic anhydrase 2	2	0.42	0.362	0.61	0.394	0.03	0.058	0.85	0.503
gi|70167127	CAH6_HUMAN	Carbonic anhydrase 6 isoform 1 precursor	117	5.30	0.014	1.53	0.182	0.77	0.912	0.47	0.020
gi|22202611	CBPD_HUMAN	Carboxypeptidase D isoform 1 precursor	2	1.05	0.850	1.21	0.360	1.02	0.916	1.12	0.676
gi|4503009	CBPE_HUMAN	Carboxypeptidase E preproprotein	4	1.02	0.875	0.88	0.809	0.66	0.133	1.05	0.725
gi|6912286	CASPE_HUMAN	Caspase-14 precursor	2	2.31	0.386	0.30	0.100	2.15	0.422	0.22	0.721

gi|4557014	CATA_HUMAN	Catalase	4	0.23	0.145	0.66	0.267	0.44	0.229	0.80	0.969
gi|4503139	CATB_HUMAN	Cathepsin B preproprotein	3	0.41	0.264	0.22	0.393	0.23	0.309	0.72	0.946
gi|4503143	CATD_HUMAN	Cathepsin D preproprotein	12	0.76	0.865	0.28	0.003	0.92	0.915	0.74	0.631
gi|6042196	CATF_HUMAN	Cathepsin F precursor	1	1.82	0.366	1.38	0.552	1.19	0.722	87.90	0.019
gi|4503149	CATG_HUMAN	Cathepsin G preproprotein	3	0.77	0.692	1.12	0.807	0.65	0.610	1.33	0.655
gi|4503155	CATL1_HUMAN	Cathepsin L1 isoform 1 preproprotein	3	0.95	0.721	1.42	0.488	0.76	0.755	0.71	0.953
gi|5174411	CD5L_HUMAN	CD5 antigen-like precursor	1	0.79	0.672	1.45	0.508	2.86	0.225	0.64	0.462
gi|10835165	CD59_HUMAN	CD59 glycoprotein preproprotein	2	1.16	0.757	1.32	0.596	1.79	0.372	0.96	0.957
gi|4502693	CD9_HUMAN	CD9 antigen	2	11.80	0.170	26.06	0.136	2.54	0.306	0.28	0.589
gi|4503029	RABP2_HUMAN	Cellular retinoic acid-binding protein 2	1	0.64	0.463	1.46	0.503	1.51	0.472	0.76	0.621
gi|5729772	CLN5_HUMAN	Ceroid-lipofuscinosis neuronal protein 5	1	1.57	0.446	0.30	0.202	0.11	0.113	0.44	0.288
gi|4557485	CERU_HUMAN	Ceruloplasmin precursor	10	0.30	0.529	0.62	0.509	0.54	0.683	0.84	0.967
gi|68533253	CH3L2_HUMAN	Chitinase-3-like protein 2 isoform b	1	0.83	0.695	0.89	0.939	0.82	0.581	0.85	0.703
gi|14251209	CLIC1_HUMAN	Chloride intracellular channel protein 1	2	7.05	0.569	7.80	0.500	0.95	0.953	2.03	0.681
gi|355594753	CLUS_HUMAN	Clusterin preproprotein	11	1.60	0.703	0.94	0.892	0.79	0.591	1.51	0.646
gi|21624607	COTL1_HUMAN	Coactosin-like protein	3	0.77	0.338	0.92	0.006	1.25	0.272	1.24	0.314
gi|109134349	COPG2_HUMAN	Coatomer subunit gamma-2	1	0.48	0.314	0.27	0.184	0.01	0.063	87.90	0.017
gi|37622887	EST2_HUMAN	Cocaine esterase isoform 2	4	0.96	0.714	1.24	0.853	0.63	0.304	1.13	0.506
gi|5031635	COF1_HUMAN	Cofilin-1	3	1.46	0.611	7.18	0.072	1.28	0.538	1.98	0.222
gi|55743096	COEA1_HUMAN	Collagen alpha-1(XIV) chain precursor	4	2.01	0.246	1.21	0.654	1.13	0.700	1.17	0.647
gi|62739183	C1QT3_HUMAN	Complement C1q tumor necrosis factor-related protein 3 isoform b precursor	1	2.07	0.311	0.69	0.525	1.24	0.677	1.19	0.719
gi|115298678	CO3_HUMAN	Complement C3 precursor	49	1.27	0.420	1.80	0.531	1.12	0.794	0.77	0.304
gi|67190748	CO4A_HUMAN	Complement C4-A isoform 1 preproprotein	14	1.71	0.214	1.14	0.797	1.26	0.851	0.77	0.486
gi|67782358	CFAB_HUMAN	Complement factor B preproprotein	6	0.87	0.713	0.63	0.167	2.96	0.955	1.09	0.984
gi|62739186	CFAH_HUMAN	Complement factor H isoform a precursor	4	1.15	0.937	1.02	0.685	2.33	0.396	0.99	0.417
gi|5902134	COR1A_HUMAN	Coronin-1A	1	0.65	0.475	4.57	0.433	0.65	0.667	2.81	0.375

gi|4885165	CYTA_HUMAN	Cystatin-A	13	8.63	0.004	13.93	0.002	1.57	0.066	1.51	0.912
gi|4503117	CYTB_HUMAN	Cystatin-B	111	0.92	0.995	0.83	0.964	0.35	0.971	1.18	0.825
gi|4503107	CYTC_HUMAN	Cystatin-C precursor	39	1.53	0.260	0.61	0.834	0.41	0.656	0.35	0.222
gi|19882256	CYTD_HUMAN	Cystatin-D precursor	66	3.84	0.293	1.12	0.850	0.72	0.466	1.04	0.912
gi|4503109	CYTS_HUMAN	Cystatin-S precursor	307	0.32	0.172	0.38	0.745	0.32	0.419	0.77	0.849
gi|4503105	CYTT_HUMAN	Cystatin-SA precursor	151	0.84	0.475	0.59	0.473	0.41	0.777	0.61	0.766
gi|19882251	CYTN_HUMAN	Cystatin-SN precursor	387	1.24	0.765	0.65	0.208	1.16	0.986	0.21	0.689
gi|300244562	CRIS3_HUMAN	Cysteine-rich secretory protein 3 isoform 2 precursor	22	0.88	0.310	1.79	0.372	0.59	0.464	1.07	0.578
gi|11386157	CDD_HUMAN	Cytidine deaminase	1	0.10	0.243	0.22	0.712	7.73	0.779	0.42	0.540
gi|4502985	CX6B1_HUMAN	Cytochrome c oxidase subunit 6B1	1	0.70	0.551	0.70	0.487	0.70	0.511	0.82	0.902
gi|271398239	CNDP2_HUMAN	Cytosolic non-specific dipeptidase isoform 1	1	0.78	0.652	0.40	0.260	0.82	0.704	0.86	0.781
gi|4503291	DOPD_HUMAN	D-dopachrome decarboxylase	1	1.84	0.543	0.72	0.961	5.97	0.215	0.08	0.354
gi|148539844	DMBT1_HUMAN	Deleted in malignant brain tumors 1 protein isoform c precursor	135	1.91	0.023	0.97	0.858	2.01	0.051	1.13	0.737
gi|116235485	DNER_HUMAN	Delta and Notch-like epidermal growth factor-related receptor precursor	1	1.18	0.735	0.21	0.158	1.36	0.569	1.16	0.758
gi|13435366	DSC2_HUMAN	Desmocollin-2 isoform Dsc2b preproprotein	23	0.82	0.617	0.72	0.423	0.98	0.622	0.89	0.112
gi|148539848	DSC3_HUMAN	Desmocollin-3 isoform Dsc3b preproprotein	5	0.70	0.262	0.95	0.616	1.15	0.715	3.98	0.488
gi|119703744	DSG1_HUMAN	Desmoglein-1 preproprotein	14	0.51	0.004	0.70	0.201	0.94	0.740	1.38	0.468
gi|119964718	DSG3_HUMAN	Desmoglein-3 preproprotein	16	1.34	0.298	1.71	0.368	0.69	0.440	0.30	0.138
gi|4758092	DIAC_HUMAN	Di-N-acetylchitobiase precursor	1	0.82	0.597	1.00	0.955	2.83	0.122	0.96	0.957
gi|62420888	DPP2_HUMAN	Dipeptidyl peptidase 2 preproprotein	1	1.16	0.736	1.28	0.545	0.65	0.296	0.56	0.300
gi|18491024	DPP3_HUMAN	Dipeptidyl peptidase 3 isoform 1	3	1.69	0.400	6.19	0.134	0.65	0.473	0.07	0.095
gi|4501915	ADAM9_HUMAN	Disintegrin and metalloproteinase domain-containing protein 9 precursor	1	0.72	0.598	2.01	0.374	0.93	0.987	2.29	0.315
gi|82659087	STAU1_HUMAN	Double-stranded RNA-binding protein Staufen homolog 1 isoform b	2	2.23	0.276	0.12	0.236	0.19	0.105	3.94	0.166
gi|294997282	DAG1_HUMAN	Dystroglycan preproprotein	5	0.86	0.836	0.34	0.031	0.67	0.669	0.70	0.411
gi|30240932	EHD1_HUMAN	EH domain-containing protein 1	1	1.32	0.600	1.45	0.514	0.77	0.642	1.60	0.435
gi|4505787	ELAF_HUMAN	Elafin preproprotein	1	2.96	0.221	2.07	0.317	1.63	0.431	1.27	0.618

gi|4503471	EF1A1_HUMAN	Elongation factor 1-alpha 1	4	1.21	0.427	1.17	0.524	1.00	0.695	0.93	0.539
gi|4503477	EF1B_HUMAN	Elongation factor 1-beta	1	0.86	0.779	0.69	0.517	2.49	0.255	1.00	0.984
gi|4503481	EF1G_HUMAN	Elongation factor 1-gamma	2	1.53	0.471	0.91	0.868	0.60	0.418	2.13	0.302
gi|4503483	EF2_HUMAN	Elongation factor 2	2	0.58	0.311	0.55	0.274	0.65	0.388	0.67	0.407
gi|94818901	ERAP1_HUMAN	Endoplasmic reticulum aminopeptidase 1 isoform a precursor	2	1.10	0.842	1.82	0.363	1.04	0.932	3.53	0.191
gi|45243507	ECP_HUMAN	Eosinophil cationic protein precursor	1	1.71	0.798	0.95	0.770	1.57	0.851	1.21	0.490
gi|7657069	ERO1A_HUMAN	ERO1-like protein alpha precursor	2	1.63	0.199	0.94	0.891	0.91	0.879	0.36	0.258
gi|38016911	STOM_HUMAN	Erythrocyte band 7 integral membrane protein isoform a	1	1.64	0.416	1.38	0.551	1.53	0.471	1.26	0.650
gi|15187164	LACRT_HUMAN	Extracellular glycoprotein lacritin precursor	1	0.59	0.740	0.34	0.668	2.19	0.428	1.46	0.669
gi|322302700	ECM1_HUMAN	Extracellular matrix protein 1 isoform 3 precursor	6	1.17	0.161	1.32	0.184	0.85	0.589	0.79	0.497
gi|118582275	SODE_HUMAN	Extracellular superoxide dismutase [Cu-Zn] precursor	1	2.11	0.306	3.34	0.198	0.36	0.232	46.13	0.020
gi|21614499	EZRI_HUMAN	Ezrin	12	2.15	0.835	1.94	0.449	0.53	0.032	1.18	0.450
gi|5453597	CAZA1_HUMAN	F-actin-capping protein subunit alpha-1	3	1.58	0.969	3.53	0.466	0.82	0.597	1.58	0.654
gi|330864679	CAPZB_HUMAN	F-actin-capping protein subunit beta isoform 2	6	0.80	0.100	1.02	0.998	0.90	0.304	1.12	0.416
gi|4557581	FABP5_HUMAN	Fatty acid-binding protein, epidermal	21	0.38	0.179	0.38	0.042	0.11	0.020	0.93	0.679
gi|41281905	URP2_HUMAN	Fermitin family homolog 3 long form	1	3.13	0.208	0.69	0.525	1.57	0.449	1.72	0.393
gi|11761629	FIBA_HUMAN	Fibrinogen alpha chain isoform alpha preproprotein	2	5.75	0.029	6.19	0.014	0.60	0.478	1.01	0.960
gi|70906435	FIBB_HUMAN	Fibrinogen beta chain isoform 1 preproprotein	11	1.34	0.647	0.67	0.192	0.94	0.668	1.41	0.212
gi|70906439	FIBG_HUMAN	Fibrinogen gamma chain isoform gamma-B precursor	8	1.26	0.415	1.47	0.353	1.45	0.425	1.10	0.739
gi|71040111	FMOD_HUMAN	Fibromodulin precursor	1	1.08	0.908	1.16	0.768	1.32	0.600	1.07	0.946
gi|47132557	FINC_HUMAN	Fibronectin isoform 1 preproprotein	16	1.80	0.522	1.27	0.479	0.36	0.059	0.59	0.772
gi|62122917	FILA2_HUMAN	Filaggrin-2	1	1.18	0.729	1.22	0.686	3.25	0.203	2.38	0.268
gi|160420317	FLNA_HUMAN	Filamin-A isoform 2	6	1.02	0.527	1.42	0.620	2.75	0.326	2.31	0.244
gi|4502419	BLVRB_HUMAN	Flavin reductase (NADPH)	2	0.97	0.965	1.61	0.304	0.61	0.301	1.31	0.506
gi|4758400	FOLR1_HUMAN	Folate receptor alpha precursor	3	1.32	0.599	0.90	0.855	0.68	0.511	0.95	0.926
gi|4557305	ALDOA_HUMAN	Fructose-bisphosphate aldolase A isoform 1	13	1.12	0.404	1.91	0.421	0.56	0.183	1.66	0.037

gi|4885063	ALDOC_HUMAN	Fructose-bisphosphate aldolase C	2	1.27	0.540	1.17	0.969	1.96	0.323	1.50	0.442
gi|4557587	FAAA_HUMAN	Fumarylacetoacetase	1	1.18	0.734	6.98	0.126	1.98	0.328	1.92	0.338
gi|4505579	FURIN_HUMAN	Furin preproprotein	5	0.78	0.673	0.78	0.621	0.67	0.456	3.94	0.452
gi|296317297	AGRG2_HUMAN	G-protein coupled receptor 64 isoform 6 precursor	1	0.22	0.526	0.29	0.301	1.39	0.664	0.62	0.361
gi|115430223	LEG3_HUMAN	Galectin-3 isoform 1	2	0.74	0.849	0.57	0.315	0.86	0.831	1.33	0.505
gi|5031863	LG3BP_HUMAN	Galectin-3-binding protein precursor	9	1.63	0.961	0.69	0.467	0.94	0.836	0.43	0.834
gi|4504985	LEG7_HUMAN	Galectin-7	2	1.12	0.662	0.90	0.479	1.02	0.824	0.80	0.560
gi|39995109	SAP3_HUMAN	Ganglioside GM2 activator isoform 1 precursor	1	1.19	0.726	0.93	0.890	0.51	0.337	1.42	0.524
gi|4504165	GELS_HUMAN	Gelsolin isoform a precursor	16	0.67	0.614	0.62	0.678	1.07	0.692	1.25	0.108
gi|13435377	GMEB1_HUMAN	Glucocorticoid modulatory element-binding protein 1 isoform 1	1	0.70	0.544	0.77	0.635	4.57	0.160	0.61	0.435
gi|109389365	G6PD_HUMAN	Glucose-6-phosphate 1-dehydrogenase isoform a	7	0.64	0.591	1.07	0.152	0.67	0.619	0.37	0.382
gi|18201905	G6PI_HUMAN	Glucose-6-phosphate isomerase isoform 2	17	0.47	0.861	0.67	0.853	0.42	0.054	1.46	0.064
gi|48255891	GLU2B_HUMAN	Glucosidase 2 subunit beta isoform 2 precursor	1	1.34	0.576	1.29	0.617	0.44	0.283	0.77	0.641
gi|54607043	GLCM_HUMAN	Glucosylceramidase isoform 1 precursor	2	1.03	0.938	0.71	0.549	1.24	0.677	0.70	0.547
gi|4504025	GLRX1_HUMAN	Glutaredoxin-1	1	0.82	0.709	0.48	0.317	1.47	0.493	6.73	0.128
gi|4758484	GSTO1_HUMAN	Glutathione S-transferase omega-1 isoform 1	2	1.77	0.420	8.55	0.033	0.75	0.732	0.89	0.692
gi|4504183	GSTP1_HUMAN	Glutathione S-transferase P	25	0.37	0.215	0.66	0.436	0.46	0.868	1.49	0.235
gi|7669492	G3P_HUMAN	Glyceraldehyde-3-phosphate dehydrogenase isoform 1	43	0.74	0.914	1.43	0.216	0.47	0.164	1.39	0.120
gi|21361370	PYGB_HUMAN	Glycogen phosphorylase, brain form	5	0.61	0.357	0.72	0.273	0.42	0.179	0.58	0.245
gi|71037379	PYGL_HUMAN	Glycogen phosphorylase, liver form isoform 1	10	1.49	0.247	0.96	0.681	0.55	0.157	0.83	0.390
gi|7705987	GLTP_HUMAN	Glycolipid transfer protein	1	0.19	0.150	0.89	0.832	1.47	0.497	2.91	0.221
gi|29550838	GOLM1_HUMAN	Golgi membrane protein 1	4	0.74	0.239	0.55	0.056	0.72	0.426	0.70	0.571
gi|11641247	GAPR1_HUMAN	Golgi-associated plant pathogenesis-related protein 1	1	0.66	0.488	1.12	0.810	1.53	0.470	1.34	0.581
gi|4504151	GRN_HUMAN	Granulins precursor	1	1.26	0.645	1.21	0.695	0.22	0.162	1.16	0.754
gi|5453555	RAN_HUMAN	GTP-binding nuclear protein Ran	1	0.69	0.525	0.37	0.240	0.89	0.820	2.68	0.239
gi|4504041	GNAI2_HUMAN	Guanine nucleotide-binding protein G(i) subunit alpha-2 isoform 1	5	1.56	0.377	1.09	0.987	6.03	0.064	1.21	0.345

gi|157671915	GBP6_HUMAN	Guanylate-binding protein 6	1	1.00	0.980	1.20	0.704	1.27	0.641	0.95	0.936
gi|4826762	HPT_HUMAN	Haptoglobin isoform 1 preproprotein	16	1.67	0.813	0.74	0.276	0.36	0.213	1.00	0.686
gi|124256496	HS71L_HUMAN	Heat shock 70 kDa protein 1-like	25	1.36	0.569	1.41	0.537	0.59	0.410	1.50	0.479
gi|167466173	HS71B_HUMAN	Heat shock 70 kDa protein 1A/1B	42	1.08	0.242	1.47	0.876	1.46	0.290	1.27	0.422
gi|38327039	HSP74_HUMAN	Heat shock 70 kDa protein 4	2	0.63	0.200	1.41	0.912	1.21	0.508	1.28	0.622
gi|5729877	HSP7C_HUMAN	Heat shock cognate 71 kDa protein isoform 1	34	3.50	0.338	0.97	0.435	0.84	0.312	1.34	0.147
gi|4504517	HSPB1_HUMAN	Heat shock protein beta-1	7	1.41	0.496	0.74	0.911	0.59	0.421	1.69	0.695
gi|153792590	HS90A_HUMAN	Heat shock protein HSP 90-alpha isoform 1	6	1.19	0.327	1.16	0.941	1.17	0.974	2.58	0.085
gi|7705877	HN1_HUMAN	Hematological and neurological expressed 1 protein isoform 1	1	1.49	0.466	0.49	0.338	0.59	0.407	1.36	0.569
gi|7657603	HEBP2_HUMAN	Heme-binding protein 2	4	0.47	0.419	2.33	0.532	1.12	0.960	3.60	0.199
gi|4504345	HBA_HUMAN	Hemoglobin subunit alpha	8	1.53	0.503	0.18	0.397	0.02	0.014	0.79	0.376
gi|4504349	HBB_HUMAN	Hemoglobin subunit beta	28	1.09	0.852	0.73	0.827	0.07	0.159	0.41	0.972
gi|11321561	HEMO_HUMAN	Hemopexin precursor	13	2.29	0.287	0.70	0.662	0.93	0.776	0.42	0.266
gi|73858566	HEP2_HUMAN	Heparin cofactor 2 precursor	1	1.06	0.792	1.58	0.555	0.86	0.743	0.82	0.828
gi|14165437	HNRPK_HUMAN	Heterogeneous nuclear ribonucleoprotein K isoform a	2	0.63	0.626	4.53	0.601	0.58	0.448	0.85	0.883
gi|194097330	HXK3_HUMAN	Hexokinase-3	6	0.49	0.652	1.98	0.823	1.00	0.814	2.68	0.060
gi|4504425	HMGB1_HUMAN	High mobility group protein B1	2	1.34	0.583	0.80	0.682	0.80	0.679	0.37	0.239
gi|4504529	HIS1_HUMAN	Histatin-1 precursor	11	2.91	0.423	1.58	0.660	3.10	0.066	1.14	0.617
gi|4504489	HRG_HUMAN	Histidine-rich glycoprotein precursor	1	0.72	0.470	1.39	0.554	1.02	0.858	0.86	0.523
gi|15617199	H2A3_HUMAN	Histone H2A type 3	3	1.13	0.718	3.63	0.669	1.36	0.841	0.36	0.324
gi|4504259	H2B1L_HUMAN	Histone H2B type 1-L	4	0.41	0.348	0.69	0.186	0.24	0.300	0.04	0.227
gi|4504301	H4_HUMAN	Histone H4	7	0.71	0.700	0.42	0.220	0.51	0.235	0.33	0.372
gi|62912479	HLAE_HUMAN	HLA class I histocompatibility antigen, alpha chain E precursor	2	1.21	0.879	0.46	0.747	0.64	0.784	0.56	0.556
gi|19923193	F10A1_HUMAN	HSC70-interacting protein	2	0.94	0.723	3.98	0.353	1.28	0.483	2.54	0.357
gi|321117267	S4R3N1_HUMAN	HSPE1-MOB4 protein	2	3.02	0.240	0.55	0.384	2.01	0.346	0.01	0.067
gi|296179427	HCDH_HUMAN	hydroxyacyl-coenzyme A dehydrogenase, mitochondrial isoform 1 precursor	1	1.01	0.974	1.87	0.350	1.39	0.548	0.33	0.221

gi|5453832	HYOU1_HUMAN	hypoxia up-regulated protein 1 precursor	2	1.84	0.361	0.27	0.188	0.97	0.964	0.56	0.384
gi|154146262	FCGBP_HUMAN	IgGFc-binding protein precursor	45	1.43	0.149	1.42	0.672	1.01	0.334	0.86	0.016
gi|21489959	IGJ_HUMAN	Immunoglobulin J chain precursor	6	1.02	0.886	1.07	0.904	1.57	0.724	1.13	0.957
gi|372466586	IGLL5_HUMAN	Immunoglobulin lambda-like polypeptide 5 isoform 2	113	1.27	0.264	1.24	0.890	0.96	0.262	0.57	0.577
gi|19923142	IMB1_HUMAN	Importin subunit beta-1	3	0.21	0.721	0.81	0.999	0.80	0.771	1.13	0.630
gi|4504703	INPP_HUMAN	Inositol polyphosphate 1-phosphatase	1	0.61	0.368	2.51	0.290	1.96	0.274	1.53	0.942
gi|11527402	ITM2B_HUMAN	Integral membrane protein 2B	1	0.80	0.528	1.71	0.553	0.15	0.450	0.53	0.529
gi|156119625	ITIH1_HUMAN	Inter-alpha-trypsin inhibitor heavy chain H1 isoform a precursor	3	0.53	0.358	0.40	0.259	0.38	0.244	1.49	0.490
gi|31542984	ITIH4_HUMAN	Inter-alpha-trypsin inhibitor heavy chain H4 isoform 1 precursor	4	0.84	0.658	0.40	0.353	0.74	0.537	0.83	0.784
gi|27894317	IL1RA_HUMAN	Interleukin-1 receptor antagonist protein isoform 2	13	0.67	0.530	0.88	0.728	0.14	0.341	2.13	0.304
gi|4504653	IL18_HUMAN	Interleukin-18 isoform 1 proprotein	2	0.90	0.621	0.87	0.281	0.81	0.458	0.94	0.652
gi|7657092	IL36A_HUMAN	Interleukin-36 alpha	4	0.70	0.705	1.38	0.313	0.47	0.192	0.41	0.479
gi|9665234	IL36G_HUMAN	Interleukin-36 gamma	1	0.20	0.292	0.27	0.234	0.14	0.193	2.05	0.048
gi|28610147	IL6RB_HUMAN	Interleukin-6 receptor subunit beta isoform 1 precursor	2	1.05	0.813	0.73	0.998	1.25	0.493	1.27	0.511
gi|44890059	INVO_HUMAN	Involucrin	10	2.31	0.309	1.57	0.947	4.79	0.345	0.68	0.456
gi|28178825	IDHC_HUMAN	Isocitrate dehydrogenase [NADP] cytoplasmic	5	0.64	0.419	0.57	0.314	0.57	0.511	0.93	0.783
gi|46852147	SYIM_HUMAN	Isoleucine--tRNA ligase, mitochondrial precursor	2	0.79	0.640	0.82	0.684	0.99	0.992	1.31	0.627
gi|4504875	KLK1_HUMAN	Kallikrein-1 preproprotein	17	9.82	0.067	2.09	0.155	5.92	0.113	0.34	0.220
gi|5803199	KLK11_HUMAN	Kallikrein-11 isoform 1 precursor	1	0.82	0.783	0.70	0.704	1.06	0.849	0.65	0.509
gi|11496281	KLK13_HUMAN	Kallikrein-13 precursor	3	1.21	0.495	1.26	0.472	0.38	0.182	1.06	0.928
gi|61744426	KLK6_HUMAN	Kallikrein-6 isoform B	2	0.46	0.779	0.77	0.878	0.92	0.932	0.60	0.773
gi|4826950	KLK7_HUMAN	Kallikrein-7 isoform 1 preproprotein	1	0.84	0.900	1.05	0.588	0.83	0.743	1.89	0.345
gi|131412225	K1C13_HUMAN	Keratin, type I cytoskeletal 13 isoform a	15	1.67	0.431	0.72	0.666	3.53	0.037	2.78	0.802
gi|15431310	K1C14_HUMAN	Keratin, type I cytoskeletal 14	16	1.04	0.153	0.25	0.184	0.41	0.011	1.47	0.447
gi|24430192	K1C16_HUMAN	Keratin, type I cytoskeletal 16	14	2.17	0.181	0.92	0.260	0.16	0.135	0.86	0.038
gi|55956899	K1C9_HUMAN	Keratin, type I cytoskeletal 9	9	1.20	0.664	1.69	0.973	3.02	0.054	1.45	0.274

gi|119395750	K2C1_HUMAN	Keratin, type II cytoskeletal 1	43	2.68	0.231	0.65	0.731	1.74	0.300	2.19	0.202
gi|47132620	K22E_HUMAN	Keratin, type II cytoskeletal 2 epidermal	18	4.13	0.252	99.08	0.085	1.42	0.556	0.24	0.179
gi|153791670	K22O_HUMAN	Keratin, type II cytoskeletal 2 oral	10	1.08	0.863	0.40	0.112	5.35	0.034	0.75	0.497
gi|119395754	K2C5_HUMAN	Keratin, type II cytoskeletal 5	15	1.45	0.361	1.38	0.451	0.75	0.512	2.00	0.178
gi|156231037	KNG1_HUMAN	Kininogen-1 isoform 1 precursor	2	1.53	0.647	1.05	0.571	1.74	0.522	0.41	0.323
gi|5031857	LDHA_HUMAN	L-lactate dehydrogenase A chain isoform 1	15	0.70	0.659	3.53	0.070	0.93	0.980	1.43	0.520
gi|4557032	LDHB_HUMAN	L-lactate dehydrogenase B chain	3	0.55	0.307	1.14	0.754	1.64	0.341	1.26	0.593
gi|262206315	LYAM1_HUMAN	L-selectin precursor	1	0.65	0.477	1.06	0.892	1.89	0.347	1.39	0.546
gi|40549418	PERL_HUMAN	Lactoperoxidase isoform 1 preproprotein	52	1.02	0.770	0.86	0.816	0.69	0.552	0.48	0.330
gi|54607120	TRFL_HUMAN	Lactotransferrin isoform 1 precursor	115	1.33	0.254	1.63	0.240	3.40	0.024	0.52	0.650
gi|118402586	LGUL_HUMAN	Lactoylglutathione lyase	1	1.18	0.727	1.57	0.450	1.27	0.636	1.69	0.404
gi|16418467	A2GL_HUMAN	Leucine-rich alpha-2-glycoprotein precursor	5	1.38	0.572	1.37	0.797	0.76	0.529	0.92	0.895
gi|13489087	ILEU_HUMAN	Leukocyte elastase inhibitor	20	1.46	0.326	3.91	0.105	0.65	0.903	0.88	0.381
gi|4505029	LKHA4_HUMAN	Leukotriene A-4 hydrolase isoform 1	11	0.89	0.883	2.03	0.301	0.87	0.920	1.75	0.139
gi|4504963	LCN1_HUMAN	Lipocalin-1 isoform 1 precursor	13	2.01	0.832	3.60	0.797	0.44	0.871	0.42	0.300
gi|68508967	EST1_HUMAN	Liver carboxylesterase 1 isoform a precursor	9	0.63	0.035	0.91	0.106	0.25	0.014	0.91	0.739
gi|50726979	FCG3A_HUMAN	Low affinity immunoglobulin gamma Fc region receptor III-A isoform a precursor	2	0.82	0.756	0.75	0.702	0.95	0.917	0.84	0.945
gi|4505047	LUM_HUMAN	Lumican precursor	2	1.47	0.266	1.06	0.493	1.58	0.247	1.05	0.623
gi|93004088	LYPD3_HUMAN	Ly6/PLAUR domain-containing protein 3 precursor	5	0.74	0.546	0.65	0.357	0.78	0.568	0.95	0.881
gi|7669503	LAMP2_HUMAN	Lysosome-associated membrane glycoprotein 2 isoform B precursor	1	3.19	0.345	2.15	0.540	1.06	0.927	0.98	0.872
gi|4557894	LYSC_HUMAN	Lysozyme C precursor	33	0.46	0.622	0.47	0.180	3.31	0.158	1.31	0.300
gi|4505185	MIF_HUMAN	Macrophage migration inhibitory factor	6	1.06	0.987	1.14	0.890	0.35	0.562	0.82	0.759
gi|5174539	MDHC_HUMAN	Malate dehydrogenase, cytoplasmic isoform 2	3	1.92	0.356	1.64	0.425	0.50	0.712	1.13	0.510
gi|21735621	MDHM_HUMAN	Malate dehydrogenase, mitochondrial precursor	7	0.96	0.889	0.92	0.870	0.73	0.685	1.28	0.434
gi|31542650	MANS1_HUMAN	MANSC domain-containing protein 1 precursor	2	0.65	0.485	0.84	0.747	0.73	0.576	1.34	0.584
gi|299890879	MGP_HUMAN	Matrix Gla protein isoform 1 precursor	2	1.01	0.976	1.04	0.921	3.02	0.216	0.44	0.288

gi|74272287	MMP9_HUMAN	Matrix metalloproteinase-9 preproprotein	9	0.22	0.224	1.61	0.830	0.54	0.419	1.10	0.576
gi|134244281	TRFM_HUMAN	Melanotransferrin isoform 1 precursor	6	1.29	0.617	0.77	0.630	0.41	0.265	0.90	0.840
gi|53988380	MSLN_HUMAN	Mesothelin isoform 2 preproprotein	5	2.29	0.792	1.75	0.438	1.20	0.345	0.31	0.467
gi|4507509	TIMP1_HUMAN	Metalloproteinase inhibitor 1 precursor	8	1.18	0.832	1.50	0.778	0.87	0.749	0.54	0.103
gi|4505257	MOES_HUMAN	Moesin	9	0.14	0.271	0.63	0.908	0.50	0.485	2.94	0.287
gi|4557417	CD14_HUMAN	Monocyte differentiation antigen CD14 precursor	4	1.38	0.347	1.89	0.712	0.61	0.283	0.65	0.346
gi|161016767	MUC21_HUMAN	Mucin-21 precursor	1	1.12	0.536	1.39	0.514	1.56	0.352	0.96	0.972
gi|112382231	MUC4_HUMAN	Mucin-4 isoform e precursor	2	1.33	0.786	0.53	0.487	1.27	0.295	0.41	0.394
gi|301172750	MUC5B_HUMAN	Mucin-5B precursor	309	1.01	0.145	1.00	0.166	1.02	0.045	0.91	0.005
gi|222418645	MUC7_HUMAN	Mucin-7 precursor	4	5.35	0.703	1.69	0.521	1.00	0.455	0.79	0.653
gi|71361688	PRTN3_HUMAN	Myeloblastin precursor	13	6.37	0.028	6.03	0.028	1.57	0.194	0.90	0.609
gi|4505227	MNDA_HUMAN	Myeloid cell nuclear differentiation antigen	1	0.40	0.261	4.97	0.152	1.19	0.718	0.86	0.775
gi|4557759	PERM_HUMAN	Myeloperoxidase precursor	6	0.67	0.483	0.47	0.543	0.93	0.422	0.86	0.701
gi|88999583	MYL6_HUMAN	Myosin light polypeptide 6 isoform 2	2	1.18	0.700	1.09	0.836	1.20	0.644	1.66	0.457
gi|5453740	ML12A_HUMAN	Myosin regulatory light chain 12A	1	7.73	0.272	11.59	0.297	2.51	0.575	1.31	0.620
gi|12667788	MYH9_HUMAN	Myosin-9	16	0.59	0.334	1.28	0.610	0.72	0.083	1.66	0.278
gi|157502212	GALT7_HUMAN	N-acetylgalactosaminyltransferase 7	2	0.96	0.958	1.10	0.841	2.01	0.320	1.49	0.488
gi|14249738	GNPTG_HUMAN	N-acetylglucosamine-1-phosphotransferase subunit gamma precursor	1	0.91	0.978	0.85	0.587	0.86	0.575	1.03	0.894
gi|5802984	B4GA1_HUMAN	N-acetyllactosaminide beta-1,3-N-acetylglucosaminyltransferase	1	0.79	0.698	1.00	0.986	0.96	0.892	1.24	0.671
gi|70995396	NQO1_HUMAN	NAD(P)H dehydrogenase [quinone] 1 isoform b	1	0.76	0.621	0.52	0.344	0.01	0.046	0.97	0.965
gi|333033787	NACA_HUMAN	Nascent polypeptide-associated complex subunit alpha isoform a	2	0.98	0.331	1.05	0.130	0.99	0.552	1.13	0.109
gi|4826904	NEUS_HUMAN	Neuroserpin precursor	2	0.63	0.396	1.31	0.578	1.33	0.527	1.09	0.842
gi|88900491	GANAB_HUMAN	Neutral alpha-glucosidase AB isoform 3 precursor	1	0.93	0.901	0.70	0.358	2.31	0.150	1.20	0.592
gi|4505221	MMP8_HUMAN	Neutrophil collagenase preproprotein	4	1.11	0.805	1.15	0.992	1.77	0.528	0.67	0.166
gi|67189970	NCF2_HUMAN	Neutrophil cytosol factor 2 isoform 1	1	14.45	0.271	1.58	0.973	1.96	0.506	2.65	0.431
gi|4758146	DEF1_HUMAN	Neutrophil defensin 1 preproprotein	5	10.28	0.098	1.63	0.492	1.21	0.307	1.25	0.377

gi|4503549	ELNE_HUMAN	Neutrophil elastase preproprotein	5	0.49	0.320	3.70	0.184	1.57	0.451	1.63	0.423
gi|38455402	NGAL_HUMAN	Neutrophil gelatinase-associated lipocalin precursor	15	1.94	0.021	1.60	0.599	1.58	0.136	0.85	0.889
gi|5031977	NAMPT_HUMAN	Nicotinamide phosphoribosyltransferase precursor	1	0.41	0.696	0.66	0.802	1.47	0.370	2.29	0.175
gi|66392203	NDKB_HUMAN	NME1-NME2 protein	4	1.60	0.691	0.42	0.738	0.43	0.783	0.66	0.933
gi|4506549	RNAS2_HUMAN	Non-secretory ribonuclease precursor	3	0.58	0.404	1.84	0.359	0.88	0.812	1.18	0.727
gi|47825361	FBX50_HUMAN	Non-specific cytotoxic cell receptor protein 1 homolog	1	1.47	0.706	0.83	0.964	2.05	0.652	1.15	0.772
gi|5031985	NTF2_HUMAN	Nuclear transport factor 2	1	1.32	0.640	1.12	0.737	1.66	0.412	1.12	0.934
gi|20070228	NUCB1_HUMAN	Nucleobindin-1 precursor	5	1.10	0.894	0.67	0.712	0.81	0.995	0.12	0.372
gi|4826870	NUCB2_HUMAN	Nucleobindin-2 precursor	14	0.64	0.363	0.21	0.065	0.97	0.386	1.12	0.323
gi|11968009	SIL1_HUMAN	Nucleotide exchange factor SIL1 precursor	2	0.99	0.768	1.66	0.363	1.87	0.483	1.60	0.447
gi|32313593	OLFM4_HUMAN	Olfactomedin-4 precursor	4	0.64	0.490	99.08	0.017	0.33	0.229	0.60	0.433
gi|9910460	NIT2_HUMAN	Omega-amidase NIT2	2	0.66	0.487	0.91	0.873	0.33	0.215	2.11	0.304
gi|166235148	OSTF1_HUMAN	Osteoclast-stimulating factor 1	1	0.58	0.404	0.52	0.353	1.36	0.567	1.98	0.330
gi|30425438	OAF_HUMAN	Out at first protein homolog precursor	1	0.91	0.743	1.64	0.441	0.53	0.390	0.44	0.590
gi|46276863	PTMS_HUMAN	Parathymosin	1	0.63	0.448	0.63	0.440	1.20	0.790	0.60	0.625
gi|167900484	PTX3_HUMAN	Pentraxin-related protein PTX3 precursor	2	0.87	0.793	5.81	0.139	5.30	0.146	0.89	0.827
gi|4827036	PGRP1_HUMAN	Peptidoglycan recognition protein 1 precursor	1	2.38	0.243	0.49	0.364	1.91	0.327	2.83	0.240
gi|21070984	AMD_HUMAN	Peptidyl-glycine alpha-amidating monooxygenase isoform a preproprotein	2	1.96	0.782	1.71	0.565	0.79	0.682	11.27	0.358
gi|10863927	PPIA_HUMAN	Peptidyl-prolyl cis-trans isomerase A	20	0.42	0.328	0.90	0.886	0.54	0.476	0.53	0.550
gi|4758950	PPIB_HUMAN	Peptidyl-prolyl cis-trans isomerase B precursor	12	1.89	0.405	0.79	0.239	2.00	0.806	0.21	0.089
gi|4503725	FKB1A_HUMAN	Peptidyl-prolyl cis-trans isomerase FKBP1A isoform a	3	0.39	0.789	0.17	0.408	0.72	0.838	0.79	0.753
gi|17149842	FKBP2_HUMAN	Peptidyl-prolyl cis-trans isomerase FKBP2 precursor	1	0.53	0.511	1.42	0.515	1.56	0.592	0.95	0.942
gi|255958306	PLIN3_HUMAN	Perilipin-3 isoform 3	4	1.02	0.779	0.97	0.698	0.88	0.714	0.96	0.925
gi|45439327	PEPL_HUMAN	Periplakin	4	0.81	0.351	0.65	0.876	0.97	0.837	1.89	0.259
gi|4505591	PRDX1_HUMAN	Peroxiredoxin-1	7	1.43	0.641	0.83	0.900	0.67	0.331	1.37	0.808
gi|32189392	PRDX2_HUMAN	Peroxiredoxin-2 isoform a	7	1.79	0.386	1.12	0.591	0.83	0.381	1.01	0.781

gi|5453549	PRDX4_HUMAN	Peroxiredoxin-4 precursor	6	0.82	0.064	1.12	0.125	1.31	0.004	1.07	0.235
gi|6912238	PRDX5_HUMAN	Peroxiredoxin-5, mitochondrial isoform a precursor	5	1.03	0.450	1.82	0.837	1.42	0.395	1.26	0.769
gi|4758638	PRDX6_HUMAN	Peroxiredoxin-6	3	0.97	0.794	1.74	0.235	0.68	0.355	0.51	0.481
gi|4505621	PEBP1_HUMAN	Phosphatidylethanolamine-binding protein 1 preproprotein	8	0.81	0.970	2.83	0.068	0.80	0.499	1.37	0.409
gi|21361621	PGM1_HUMAN	Phosphoglucomutase-1 isoform 1	3	0.58	0.675	0.97	0.801	1.12	0.203	1.22	0.313
gi|63055049	PGM2_HUMAN	Phosphoglucomutase-2	1	1.33	0.592	1.24	0.674	0.87	0.793	1.10	0.841
gi|4505763	PGK1_HUMAN	Phosphoglycerate kinase 1	18	0.62	0.715	1.61	0.725	0.85	0.978	1.32	0.528
gi|4505753	PGAM1_HUMAN	Phosphoglycerate mutase 1	7	0.59	0.077	0.73	0.836	1.05	0.896	1.56	0.163
gi|110227598	PLBL1_HUMAN	Phospholipase B-like 1 precursor	4	1.25	0.347	1.21	0.587	0.90	0.472	1.07	0.580
gi|5453914	PLTP_HUMAN	Phospholipid transfer protein isoform a precursor	4	3.10	0.973	5.70	0.149	1.29	0.673	0.61	0.254
gi|39725934	PEDF_HUMAN	Pigment epithelium-derived factor precursor	4	0.49	0.625	0.59	0.332	0.69	0.476	0.61	0.411
gi|4505595	PAI2_HUMAN	Plasminogen activator inhibitor 2	2	0.53	0.332	0.75	0.592	0.39	0.660	1.45	0.680
gi|167614506	PLSL_HUMAN	Plastin-2	11	0.64	0.603	1.29	0.528	1.16	0.346	1.67	0.181
gi|7549809	PLST_HUMAN	Plastin-3 isoform 1	9	1.53	0.514	0.96	0.954	1.19	0.631	1.13	0.821
gi|7657465	PDXL2_HUMAN	Podocalyxin-like protein 2 precursor	1	1.34	0.579	0.18	0.143	0.75	0.610	1.80	0.368
gi|193083112	PCBP2_HUMAN	Poly(rC)-binding protein 2 isoform f	2	0.87	0.797	0.66	0.490	0.59	0.409	0.40	0.260
gi|289063433	ENDOU_HUMAN	Poly(U)-specific endoribonuclease isoform 1 precursor	4	0.99	0.806	1.12	0.682	0.99	0.978	0.95	0.971
gi|31377806	PIGR_HUMAN	Polymeric immunoglobulin receptor precursor	234	1.34	0.700	2.00	0.654	0.79	0.144	0.86	0.855
gi|115298684	GALT6_HUMAN	Polypeptide N-acetylgalactosaminyltransferase 6	1	0.46	0.297	0.25	0.175	0.41	0.265	1.38	0.550
gi|7657162	PFD6_HUMAN	Prefoldin subunit 6	1	1.19	0.788	1.96	0.661	9.91	0.372	17.54	0.156
gi|23110955	CATH_HUMAN	Pro-cathepsin H preproprotein	2	0.50	0.314	0.59	0.446	0.97	0.978	0.99	0.948
gi|11386147	SAP_HUMAN	Prosaposin isoform a preproprotein	10	11.38	0.022	3.70	0.179	0.66	0.758	1.13	0.812
gi|4826898	PROF1_HUMAN	Profilin-1	13	1.36	0.742	3.47	0.582	0.93	0.839	1.67	0.095
gi|241982780	PDC6I_HUMAN	Programmed cell death 6-interacting protein isoform 2	3	0.44	0.293	0.63	0.818	0.79	0.603	1.82	0.646
gi|4759224	PDCD5_HUMAN	Programmed cell death protein 5	1	0.69	0.567	1.69	0.412	0.79	0.794	1.71	0.594
gi|4505821	PIP_HUMAN	Prolactin-inducible protein precursor	163	1.11	0.165	1.03	0.636	0.95	0.312	1.01	0.523

gi|124494254	PA2G4_HUMAN	Proliferation-associated protein 2G4	1	1.49	0.436	0.70	0.563	0.63	0.460	1.21	0.665
gi|154448886	PROL4_HUMAN	Proline-rich protein 4 isoform 2 precursor	10	0.59	0.585	0.42	0.249	1.01	0.972	0.84	0.738
gi|5174387	PROM1_HUMAN	Prominin-1 isoform 1 precursor	7	0.77	0.729	0.93	0.180	11.27	0.192	2.21	0.387
gi|226059133	PTGR1_HUMAN	Prostaglandin reductase 1 isoform 1	8	0.82	0.890	0.87	0.637	0.86	0.869	0.09	0.020
gi|4506153	PRSS8_HUMAN	Prostasin preproprotein	1	0.98	0.989	0.71	0.550	0.81	0.702	1.17	0.743
gi|5453990	PSME1_HUMAN	Proteasome activator complex subunit 1 isoform 1	2	2.94	0.140	1.28	0.065	0.49	0.166	0.69	0.317
gi|4506181	PSA2_HUMAN	Proteasome subunit alpha type-2	2	0.96	0.922	0.92	0.556	1.05	0.910	1.04	0.745
gi|23110942	PSA5_HUMAN	Proteasome subunit alpha type-5 isoform 1	2	0.45	0.224	1.80	0.280	1.31	0.738	3.22	0.255
gi|22538465	PSB3_HUMAN	Proteasome subunit beta type-3	2	1.47	0.857	0.39	0.643	2.15	0.447	2.49	0.313
gi|22538467	PSB4_HUMAN	Proteasome subunit beta type-4	1	1.31	0.610	0.42	0.272	3.84	0.179	2.58	0.247
gi|4502067	AMBP_HUMAN	Protein AMBP preproprotein	2	0.71	0.347	1.06	0.641	1.19	0.532	0.95	0.895
gi|7657176	CNPY2_HUMAN	Protein canopy homolog 2 isoform 1 precursor	1	0.86	0.772	1.19	0.716	0.99	0.999	0.46	0.301
gi|62198241	CUTA_HUMAN	Protein CutA isoform 1	3	9.20	0.213	1.82	0.421	2.13	0.413	1.92	0.045
gi|5031973	PDIA6_HUMAN	Protein disulfide-isomerase A6 precursor	3	0.70	0.611	0.73	0.376	0.30	0.278	0.64	0.304
gi|20070125	PDIA1_HUMAN	Protein disulfide-isomerase precursor	6	1.34	0.693	0.54	0.614	1.41	0.462	0.95	0.869
gi|46255032	FAM3B_HUMAN	Protein FAM3B isoform b	2	1.29	0.501	1.05	0.737	1.15	0.501	1.07	0.974
gi|7661714	FAM3C_HUMAN	Protein FAM3C precursor	2	0.75	0.497	0.90	0.910	2.75	0.598	2.07	0.573
gi|20270363	FAM3D_HUMAN	Protein FAM3D precursor	3	1.18	0.561	0.63	0.891	0.65	0.204	0.54	0.373
gi|42734438	FA49B_HUMAN	Protein FAM49B	1	0.80	0.593	2.01	0.213	0.73	0.482	0.89	0.836
gi|22267436	NPS3A_HUMAN	Protein NipSnap homolog 3A	1	0.05	0.217	1.51	0.586	1.05	0.947	7.38	0.250
gi|387527987	OS9_HUMAN	Protein OS-9 isoform 5 precursor	4	1.08	0.869	0.94	0.912	1.14	0.769	0.69	0.508
gi|5032057	S10AB_HUMAN	Protein S100-A11	10	0.77	0.602	1.84	0.657	0.41	0.302	0.70	0.818
gi|17933772	S10AG_HUMAN	Protein S100-A16	1	1.13	0.634	1.33	0.672	0.79	0.506	1.02	0.739
gi|5174661	S10A2_HUMAN	Protein S100-A2	4	0.93	0.873	2.15	0.862	2.65	0.398	1.80	0.645
gi|4506765	S10A4_HUMAN	Protein S100-A4	1	1.12	0.810	1.39	0.540	1.80	0.371	0.55	0.375
gi|7657532	S10A6_HUMAN	Protein S100-A6	2	0.49	0.688	0.68	0.753	0.12	0.371	1.91	0.787

gi|115298657	S10A7_HUMAN	Protein S100-A7	22	0.94	0.099	0.96	0.361	0.49	0.026	0.18	0.003
gi|28827815	S1A7A_HUMAN	Protein S100-A7A	15	0.98	0.991	0.27	0.185	0.16	0.136	0.01	0.054
gi|21614544	S10A8_HUMAN	Protein S100-A8	36	3.40	0.188	1.00	0.374	1.91	0.529	2.05	0.109
gi|4506773	S10A9_HUMAN	Protein S100-A9	53	0.44	0.052	0.69	0.455	0.84	0.117	1.87	0.660
gi|189458821	TGM3_HUMAN	Protein-glutamine gamma-glutamyltransferase E	19	1.98	0.007	1.53	0.825	1.16	0.481	1.07	0.531
gi|4507475	TGM1_HUMAN	Protein-glutamine gamma-glutamyltransferase K	1	2.31	0.193	2.15	0.259	1.13	0.776	0.40	0.163
gi|4503635	THRB_HUMAN	Prothrombin preproprotein	2	0.66	0.875	0.69	0.536	1.94	0.395	0.27	0.140
gi|157168362	PNPH_HUMAN	Purine nucleoside phosphorylase	6	1.18	0.917	2.38	0.469	1.12	0.509	0.59	0.351
gi|158937236	PSA_HUMAN	Puromycin-sensitive aminopeptidase	10	2.68	0.290	1.42	0.484	0.57	0.140	1.12	0.412
gi|83921602	S38AA_HUMAN	Putative sodium-coupled neutral amino acid transporter 10 isoform a	2	1.43	0.643	0.52	0.387	3.94	0.332	1.06	0.964
gi|33286418	KPYM_HUMAN	Pyruvate kinase isozymes M1/M2 isoform a	15	0.45	0.056	1.94	0.351	0.82	0.177	1.36	0.001
gi|4503971	GDIA_HUMAN	rab GDP dissociation inhibitor alpha	8	1.43	0.454	0.93	0.708	2.33	0.178	0.74	0.471
gi|6598323	GDIB_HUMAN	rab GDP dissociation inhibitor beta isoform 1	9	0.87	0.849	3.44	0.470	0.98	0.743	1.28	0.793
gi|4506787	IQGA1_HUMAN	ras GTPase-activating-like protein IQGAP1	3	3.34	0.198	8.24	0.117	2.65	0.242	0.32	0.210
gi|4506381	RAC2_HUMAN	ras-related C3 botulinum toxin substrate 2	1	0.86	0.722	1.41	0.981	1.03	0.586	1.85	0.542
gi|332205939	RB11A_HUMAN	ras-related protein Rab-11A isoform 2	1	0.69	0.526	1.28	0.633	0.68	0.508	0.70	0.541
gi|13569962	RAB1B_HUMAN	ras-related protein Rab-1B	2	0.35	0.491	0.25	0.991	0.66	0.610	1.43	0.617
gi|354721184	RAB5C_HUMAN	ras-related protein Rab-5C isoform b	1	1.06	0.891	0.65	0.482	2.31	0.276	80.91	0.020
gi|34147513	RAB7A_HUMAN	ras-related protein Rab-7a	6	0.88	0.400	0.81	0.649	1.05	0.725	1.09	0.960
gi|7661678	RAP1B_HUMAN	ras-related protein Rap-1b isoform 1 precursor	2	0.47	0.600	0.04	0.147	1.82	0.595	2.07	0.251
gi|15011918	RENR_HUMAN	Renin receptor precursor	2	2.07	0.311	48.31	0.064	1.66	0.414	1.47	0.495
gi|9966777	RETN_HUMAN	Resistin precursor	1	0.98	0.724	0.66	0.654	0.18	0.402	1.37	0.987
gi|21361176	AL1A1_HUMAN	Retinal dehydrogenase 1	4	1.02	0.986	0.08	0.143	1.00	0.919	1.08	0.996
gi|46255043	TIG1_HUMAN	Retinoic acid receptor responder protein 1 isoform 1 precursor	1	2.96	0.218	0.85	0.760	0.53	0.354	1.84	0.362
gi|56676393	GDIR2_HUMAN	rho GDP-dissociation inhibitor 2	1	0.86	0.789	0.90	0.835	1.42	0.529	1.24	0.677
gi|4757766	RHG01_HUMAN	rho GTPase-activating protein 1	1	6.03	0.136	0.35	0.227	1.17	0.743	1.07	0.879

gi|4506557	RNAS4_HUMAN	Ribonuclease 4 precursor	2	0.91	0.954	1.25	0.439	1.18	0.819	1.26	0.916
gi|21361547	RINI_HUMAN	Ribonuclease inhibitor	4	4.17	0.070	1.20	0.572	0.55	0.287	1.27	0.802
gi|5231228	RNT2_HUMAN	Ribonuclease T2 precursor	8	1.21	0.806	0.77	0.828	1.07	0.596	1.18	0.631
gi|7019477	HTRA2_HUMAN	Serine protease HTRA2, mitochondrial isoform 1 preproprotein	1	1.74	0.388	0.95	0.931	0.82	0.714	0.58	0.401
gi|74027261	ISK5_HUMAN	Serine protease inhibitor Kazal-type 5 isoform b precursor	12	0.77	0.056	0.61	0.081	1.13	0.445	1.42	0.417
gi|4506003	PP1A_HUMAN	Serine/threonine-protein phosphatase PP1-alpha catalytic subunit isoform 1	1	2.15	0.297	0.93	0.890	0.11	0.110	1.41	0.537
gi|4557871	TRFE_HUMAN	Serotransferrin precursor	61	0.35	0.088	0.13	0.000	0.56	0.596	0.93	0.488
gi|8393956	SPB13_HUMAN	Serpin B13	11	7.18	0.301	5.11	0.139	0.77	0.599	0.36	0.264
gi|5902072	SPB3_HUMAN	Serpin B3	29	0.56	0.633	1.05	0.096	0.22	0.004	0.50	0.064
gi|28076869	SPB4_HUMAN	Serpin B4	19	2.25	0.283	1.17	0.750	1.24	0.670	1.00	0.988
gi|167860126	SPB5_HUMAN	Serpin B5	10	0.90	0.835	1.28	0.991	0.39	0.107	0.33	0.089
gi|425876768	SPB6_HUMAN	Serpin B6 isoform d	3	11.48	0.304	0.21	0.237	0.77	0.877	2.23	0.561
gi|4502027	ALBU_HUMAN	Serum albumin preproprotein	228	0.50	0.092	0.14	0.000	0.46	0.004	0.51	0.011
gi|4506925	SH3L1_HUMAN	SH3 domain-binding glutamic acid-rich-like protein	5	0.55	0.389	0.65	0.685	2.31	0.173	3.66	0.079
gi|13775198	SH3L3_HUMAN	SH3 domain-binding glutamic acid-rich-like protein 3	6	1.77	0.991	2.58	0.936	0.51	0.237	1.82	0.315
gi|4885607	SPRR3_HUMAN	Small proline-rich protein 3	7	0.60	0.276	0.78	0.606	1.00	0.250	0.88	0.498
gi|20270339	CANT1_HUMAN	Soluble calcium-activated nucleotidase 1	2	1.85	0.553	0.84	0.783	1.79	0.705	0.51	0.608
gi|190194423	SPRL1_HUMAN	SPARC-like protein 1 precursor	8	1.75	0.749	0.98	0.382	1.67	0.325	0.38	0.242
gi|4507261	STAT_HUMAN	Statherin isoform a precursor	133	16.44	0.002	1.51	0.409	3.94	0.084	1.17	0.442
gi|24234688	GRP75_HUMAN	Stress-70 protein, mitochondrial precursor	5	0.74	0.454	1.26	0.546	0.28	0.171	0.64	0.440
gi|5729962	SMR3B_HUMAN	Submaxillary gland androgen-regulated protein 3B precursor	69	1.75	0.465	0.98	0.848	0.86	0.653	1.10	0.548
gi|13325075	QSOX1_HUMAN	Sulfhydryl oxidase 1 isoform a precursor	4	2.09	0.724	0.89	0.236	1.80	0.094	3.77	0.028
gi|4507149	SODC_HUMAN	Superoxide dismutase [Cu-Zn]	2	0.46	0.435	1.26	0.908	1.14	0.576	1.07	0.799
gi|260436922	SBSN_HUMAN	Suprabasin isoform 1 precursor	9	0.55	0.377	0.56	0.382	0.82	0.712	0.52	0.352
gi|18379349	VAT1_HUMAN	Synaptic vesicle membrane protein VAT-1 homolog	5	2.03	0.003	1.96	0.003	2.00	0.003	0.94	0.731
gi|55749515	SDCB1_HUMAN	Syntenin-1 isoform 3	3	1.42	0.365	0.98	0.959	0.63	0.335	1.13	0.716

gi|223029410	TLN1_HUMAN	Talin-1	6	0.91	0.995	0.97	0.909	0.99	0.930	1.28	0.651
gi|156627579	TETN_HUMAN	Tetranectin precursor	1	3.44	0.380	1.37	0.524	1.74	0.634	0.58	0.796
gi|21264578	TSN1_HUMAN	Tetraspanin-1	2	2.58	0.287	2.44	0.343	7.59	0.275	0.04	0.237
gi|14249348	TXD17_HUMAN	Thioredoxin domain-containing protein 17	1	0.16	0.148	0.23	0.408	0.24	0.446	0.41	0.427
gi|42794771	TXND5_HUMAN	Thioredoxin domain-containing protein 5 isoform 1 precursor	1	1.37	0.468	0.93	0.789	0.78	0.557	1.79	0.347
gi|50592994	THIO_HUMAN	Thioredoxin isoform 1	10	2.33	0.857	2.88	0.856	1.02	0.727	0.52	0.847
gi|40317626	TSP1_HUMAN	Thrombospondin-1 precursor	3	1.80	0.521	0.61	0.630	1.96	0.060	1.94	0.388
gi|4503445	TYPH_HUMAN	Thymidine phosphorylase isoform 1 proprotein	2	1.51	0.315	1.01	0.761	0.78	0.507	1.24	0.757
gi|11056061	TYB4_HUMAN	Thymosin beta-4	2	0.13	0.515	0.78	0.708	0.05	0.235	2.03	0.363
gi|7656926	TPPC3_HUMAN	Trafficking protein particle complex subunit 3 isoform 1	1	3.40	0.195	1.26	0.647	1.96	0.334	2.07	0.311
gi|5803187	TALDO_HUMAN	Transaldolase	6	0.64	0.343	0.71	0.906	1.34	0.149	2.40	0.175
gi|21071008	TCO1_HUMAN	Transcobalamin-1 precursor	20	6.61	0.029	2.17	0.135	1.05	0.845	1.22	0.219
gi|4507357	TAGL2_HUMAN	Transgelin-2	8	4.17	0.063	1.71	0.273	0.31	0.120	0.62	0.228
gi|4507521	TKT_HUMAN	Transketolase isoform 1	14	1.41	0.208	2.03	0.492	1.41	0.762	0.81	0.887
gi|98986464	TMEDA_HUMAN	Transmembrane emp24 domain-containing protein 10 precursor	1	0.24	0.170	2.38	0.267	2.29	0.278	0.65	0.478
gi|167466252	TM11A_HUMAN	Transmembrane protease serine 11A isoform 2	2	1.74	0.343	1.10	0.803	2.51	0.176	0.87	0.761
gi|4758508	TM11D_HUMAN	Transmembrane protease serine 11D	3	0.85	0.911	1.54	0.170	0.94	0.996	1.32	0.288
gi|40254871	TM11E_HUMAN	Transmembrane protease serine 11E precursor	1	0.74	0.598	1.02	0.961	0.21	0.156	1.03	0.933
gi|30089937	T132A_HUMAN	Transmembrane protein 132A isoform b precursor	1	1.22	0.690	1.46	0.503	0.93	0.889	1.03	0.942
gi|4507725	TTHY_HUMAN	Transthyretin precursor	5	2.11	0.151	0.94	0.151	5.70	0.056	0.61	0.511
gi|226529917	TPIS_HUMAN	Triosephosphate isomerase isoform 2	21	0.72	0.067	1.02	0.712	0.47	0.686	1.74	0.201
gi|5729770	TPP1_HUMAN	Tripeptidyl-peptidase 1 preproprotein	4	0.60	0.225	1.31	0.367	1.17	0.973	2.94	0.177
gi|7657649	TMOD3_HUMAN	Tropomodulin-3	1	0.86	0.626	0.98	0.915	0.95	0.968	0.92	0.937
gi|24119203	TPM3_HUMAN	Tropomyosin alpha-3 chain isoform 2	5	0.22	0.065	0.28	0.021	0.47	0.047	2.99	0.026
gi|17921989	TBA4A_HUMAN	Tubulin alpha-4A chain	6	0.51	0.674	2.63	0.204	1.60	0.462	1.29	0.618
gi|5174735	TBB4B_HUMAN	Tubulin beta-4B chain	6	0.92	0.693	0.81	0.855	1.94	0.370	1.11	0.596

gi|56676375	TPPP3_HUMAN	Tubulin polymerization-promoting protein family member 3	2	0.81	0.696	1.32	0.346	0.86	0.850	0.24	0.943
gi|4759212	TBCA_HUMAN	Tubulin-specific chaperone A	1	0.53	0.520	4.97	0.441	13.06	0.163	0.42	0.383
gi|18104993	PTN6_HUMAN	Tyrosine-protein phosphatase non-receptor type 6 isoform 3	1	1.46	0.620	1.20	0.640	0.60	0.420	0.74	0.581
gi|373432684	UB2L3_HUMAN	Ubiquitin-conjugating enzyme E2 L3 isoform 4	1	0.64	0.460	1.37	0.561	0.77	0.635	1.00	0.986
gi|23510338	UBA1_HUMAN	Ubiquitin-like modifier-activating enzyme 1	4	1.27	0.401	1.46	0.275	1.13	0.492	0.56	0.106
gi|4507813	UGDH_HUMAN	UDP-glucose 6-dehydrogenase isoform 1	1	0.73	0.634	0.75	0.573	2.21	0.710	0.95	0.994
gi|156071476	PRR27_HUMAN	Uncharacterized protein C4orf40 precursor	2	2.56	0.355	2.03	0.434	0.38	0.189	0.82	0.290
gi|33457348	MYDGF_HUMAN	UPF0556 protein C19orf10 precursor	1	0.93	0.893	1.39	0.661	0.81	0.738	0.79	0.859
gi|58219024	LEG1H_HUMAN	UPF0762 protein C6orf58 precursor	34	1.69	0.224	1.11	0.568	0.92	0.573	1.11	0.976
gi|48255966	UGPA_HUMAN	UTP-glucose-1-phosphate uridylyltransferase isoform a	2	0.79	0.773	2.51	0.368	0.99	0.883	0.60	0.284
gi|4506387	RD23B_HUMAN	UV excision repair protein RAD23 homolog B isoform 1	2	0.07	0.091	0.13	0.121	0.69	0.524	1.58	0.440
gi|19913424	VATA_HUMAN	V-type proton ATPase catalytic subunit A	2	0.63	0.833	0.88	0.799	0.89	0.779	1.24	0.603
gi|62414289	VIME_HUMAN	Vimentin	5	1.19	0.736	0.13	0.450	0.65	0.623	0.52	0.874
gi|7669550	VINC_HUMAN	Vinculin isoform meta-VCL	5	0.58	0.472	5.55	0.289	1.01	0.394	0.77	0.553
gi|21361559	VISL1_HUMAN	Visinin-like protein 1	2	1.10	0.942	0.70	0.545	1.05	0.885	0.89	0.709
gi|32483410	VTDB_HUMAN	Vitamin D-binding protein isoform 1 precursor	10	0.45	0.122	0.19	0.151	0.50	0.262	0.82	0.552
gi|88853069	VTNC_HUMAN	Vitronectin precursor	3	1.03	0.529	0.40	0.324	1.01	0.905	0.44	0.401
gi|40068485	VWA1_HUMAN	Von Willebrand factor A domain-containing protein 1 isoform 1 precursor	2	1.74	0.436	1.01	0.901	0.88	0.593	1.32	0.505
gi|56699495	WFDC2_HUMAN	WAP four-disulfide core domain protein 2 precursor	11	2.86	0.216	0.23	0.261	4.88	0.275	1.67	0.674
gi|9257257	WDR1_HUMAN	WD repeat-containing protein 1 isoform 1	4	1.00	0.962	1.18	0.756	0.65	0.796	3.73	0.127
gi|296010966	ZN185_HUMAN	Zinc finger protein 185 isoform 5	1	0.50	0.329	0.91	0.898	1.03	1.000	1.24	0.663
gi|313482812	ZN549_HUMAN	Zinc finger protein 549 isoform 1	1	1.82	0.366	1.16	0.758	2.19	0.291	0.05	0.082
gi|4502337	ZA2G_HUMAN	Zinc-alpha-2-glycoprotein precursor	111	0.84	0.723	0.51	0.339	0.73	0.387	0.94	0.444
gi|94536866	ZG16B_HUMAN	Zymogen granule protein 16 homolog B precursor	63	19.95	0.081	23.12	0.107	2.00	0.011	0.81	0.072

ACig:	After smoking Cig
BCig:	Before smoking Cig
AShCig:	After sham smoking Cig
BShCig:	Before sham smoking Cig
S:	Smokers
NS:	Non-Smokers

Table S2. Statistical models used for variables tested based on smoking status alone and status plus adjustment for age and weight of the participants.

No adjustment		Adjust for age		Adjust for weight	
**Variable**	**P-Value**	**Variable**	**P-Value**	**Variable**	**P-Value**
Fibrinogen	0,114	Fibrinogen	0,128	Fibrinogen	0,027
Cystatin A	0,226	Cystatin A	0,236	Cystatin A	0,266
sAA levels by Western	0,085	sAA.Western	0,122	sAA.Western	0,199
Cortisol	0,637	Cortisol	0,743	Cortisol	0,88

© 2021 Sinha I. et al.
